# Simvastatin Impairs Insulin Secretion by Multiple Mechanisms in MIN6 Cells

**DOI:** 10.1371/journal.pone.0142902

**Published:** 2015-11-11

**Authors:** Nagendra Yaluri, Shalem Modi, Maykel López Rodríguez, Alena Stančáková, Johanna Kuusisto, Tarja Kokkola, Markku Laakso

**Affiliations:** 1 Institute of Clinical Medicine, Internal Medicine, Faculty of Health Sciences, University of Eastern Finland, Kuopio, Finland; 2 Department of Medicine, Kuopio University Hospital, Kuopio, Finland; University of Ulster, UNITED KINGDOM

## Abstract

Statins are widely used in the treatment of hypercholesterolemia and are efficient in the prevention of cardiovascular disease. Molecular mechanisms explaining statin-induced impairment in insulin secretion remain largely unknown. In the current study, we show that simvastatin decreased glucose-stimulated insulin secretion in mouse pancreatic MIN6 β-cells by 59% and 79% (p<0.01) at glucose concentration of 5.5 mmol/l and 16.7 mmol/l, respectively, compared to control, whereas pravastatin did not impair insulin secretion. Simvastatin induced decrease in insulin secretion occurred through multiple targets. In addition to its established effects on ATP-sensitive potassium channels (p = 0.004) and voltage-gated calcium channels (p = 0.004), simvastatin suppressed insulin secretion stimulated by muscarinic M3 or GPR40 receptor agonists (Tak875 by 33%, p = 0.002; GW9508 by 77%, p = 0.01) at glucose level of 5.5 mmol/l, and inhibited calcium release from the endoplasmic reticulum. Impaired insulin secretion caused by simvastatin treatment were efficiently restored by GPR119 or GLP-1 receptor stimulation and by direct activation of cAMP-dependent signaling pathways with forskolin. The effects of simvastatin treatment on insulin secretion were not affected by the presence of hyperglycemia. Our observation of the opposite effects of simvastatin and pravastatin on glucose-stimulated insulin secretion is in agreement with previous reports showing that simvastatin, but not pravastatin, was associated with increased risk of incident diabetes.

## Introduction

Statins are inhibitors of 3-hydroxy-3-methyl-glutaryl-CoA (HMG-CoA) reductase, the rate-limiting step in the cholesterol biosynthesis. Statins are widely used in the treatment of hypercholesterolemia, and are efficient in the prevention of cardiovascular disease events. Recently several studies, including a meta-analysis of 13 statin trials with 91,140 non-diabetic participants, have suggested that statins increase the risk of type 2 diabetes [1]. This risk has been shown to be dose-dependent [2], differs between the statins, and is the lowest for pravastatin and the highest for rosuvastatin, atorvastatin and simvastatin [3].

Type 2 diabetes is caused by impaired insulin secretion and insulin resistance [4]. Glucose-stimulated insulin release from its granules requires an increase in intracellular calcium attributable to calcium influx through voltage-gated calcium channels (VGCC) that are controlled by the ATP-sensitive potassium channels (K_ATP_). Additionally, insulin secretion is regulated by acetylcholine, glucagon like peptide 1 (GLP-1) and fatty acid- or lipid-sensing receptors (GPR40, GPR119) signaling pathways. To understand the mechanisms how statin treatment increases the risk of diabetes, a systematic investigation of all major signaling pathways regulating insulin secretion is needed. Previous reports suggest that statins block VGCC and open K_ATP_ [5, 6], but the role of other receptors and signaling pathways remain largely unknown.

We characterized the mechanisms how simvastatin, a widely used statin, and pravastatin, known to have no or little diabetogenic effect, affect insulin secretion in mouse pancreatic MIN6 β-cells. Our study reports multiple new mechanisms how simvastatin impairs insulin secretion.

## Research design and methods

The list of reagents and further details on the methods described below are given in the S1 Table and S1 Appendix.

### MIN6 cell culture

Mouse pancreatic MIN6 β-cells [7] were obtained from Merja Roivainen, National Institute for Health and Welfare, Helsinki, Finland (originally obtained from Prof. Jun-ichi Miyazaki, Osaka University, Japan). The cells were cultured at 37°C in a humidified atmosphere with 5% CO_2_ in DMEM containing 25 mM glucose supplemented with 15% heat inactivated fetal bovine serum (GIBCO), 2 mM L-glutamine (Lonza) and 100 units/ml penicillin, 100 μg/ml streptomycin (Lonza), 5 μl/l β-mercaptoethanol and 3.4 g/l NaHCO_3_. See also S1 Appendix.

### Insulin Secretion Assay

MIN6 cells were washed with glucose-free KRBH (Krebs-Ringer bicarbonate HEPES Buffer) (119 mM NaCl, 4.74 mM KCl, 2.54 mM CaCl_2_, 1.19 mM MgSO_4_, 1.19 mM KH_2_PO_4_, 10 mM HEPES, 25 mM NaHCO_3_ and 0.1% BSA) at pH 7.4 thrice before incubating them in same buffer at 37°C for 1 hour. Later, cells were washed once with glucose free KRBH and pre-incubated with either 14.3 μM simvastatin or pravastatin in the presence of activators and inhibitors in KRBH for 30 minutes at 37°C. The cells were later treated with compounds in KRBH containing either 5.5 mM or 16.7 mM glucose at 37°C for 1 hour. Ca^2+^- free KRBH Buffer (135 mM NaCl, 3.6 mM KCl, 0.5 mM NaH_2_PO_4_, 0.5 mM MgCl_2_, 10 mM HEPES, 0.1 mM EGTA, 2 mM NaHCO_3_ and 0.1% BSA) at pH 7.4 was used in the insulin secretion experiments assessing the influence of the extracellular calcium. In these experiments, the cells were treated with normal KRBH with compounds for 30 minutes, washed twice with Ca^2+^- free KRBH and later treated with compounds in Ca^2+^- free KRBH for 45 minutes. KRBH buffer was collected for insulin assay and cells were washed with PBS once. Cells were lysed with RIPA buffer containing protease and phosphatase inhibitors, collected for protein estimation and western blotting and stored at -70°C until further analysis. Proteins were estimated with BCA protein assay (Pierce, Cat. No. 23225). Insulin was measured with AlphaLISA Insulin Kit (PerkinElmer, Cat. No. AL204C) according to the manufacturer’s instructions. See also S1 Appendix.

### Glucose uptake assay

MIN6 cells were washed thrice with glucose free KRBH buffer, pre-incubated for 1 hour and were later treated with 14.3 μM simvastatin in the same buffer for 30 minutes, washed once with glucose free KRBH buffer and treated with simvastatin in KRBH buffer containing 5.5 or 16.7 mM glucose for 1 hour. KRBH buffer was removed and the cells were treated with KRBH buffer containing 0.2 mM glucose and 1 μCi 2-Deoxy-D-[2,6-^3^H] glucose (PerkinElmer, Cat. No. NET549250UC) and incubated for 15 minutes at room temperature. Cells were washed with ice cold PBS while the plates were on ice. 200 μl of 0.2 N NaOH was added to each well and the plate was incubated for 90 minutes at room temperature with constant shaking. Collected samples were stored at -70°C. Optiphase 2 (PerkinElmer) was added to the samples and radioactivity was measured using 1450 MicroBeta Trilux (Wallac). See also S1 Appendix.

### Immunoblotting

After the incubation according to the specific experiment the cells were washed once with PBS and lysed with RIPA buffer along with protease and phosphatase inhibitors (Roche). Protein concentrations were measured by BCA protein assay (Pierce, Cat. No. 23225). 20 μg/lane of protein samples containing NuPAGE LDS sample buffer and reducing agent were loaded into 4–12% NuPAGE Bis-Tris gels (Life Technologies, Cat. No. NP0336BOX), subjected to gel electrophoresis and transferred to polyvinylidene fluoride (PVDF) membranes (GE Healthcare, Cat. No. RPN303F). The bands were visualized using chemiluminescence (ECL Plus, Pierce) and images were captured in Image Quant RT-ECL equipment (GE Healthcare). Bands were quantified by applying Quantity One software (Bio-Rad). Protein expressions were normalized with α-tubulin or GAPDH or actin protein levels. See also S1 Appendix.

### ADP/ATP ratio and pyruvate assay

After 1 hour of incubation with the compounds as mentioned above, samples were collected for ADP/ATP ratio and pyruvate levels using kits (Cat. No. ab65313 and ab65342, Abcam) according to the manufacturer’s instructions. See also S1 Appendix.

### cAMP and PKC assay

GPR119-overexpressing CHO-K1 cell lines (Euroscreen) were used to determine cAMP using cAMP HTRF^™^ functional assay (FAST-0370C). The cells were first treated with 14.3 μM simvastatin and pravastatin for 30 minutes and then stimulated with GPR119 agonist oleoylethanolamide, which increases cAMP levels. The total PKC activity in MIN6 cells was analyzed with PKC kinase activity kit (Enzo, Cat. No. ADI-EKS-420A) according to the manufacturer’s instructions.

### Intracellular calcium measurements

Intracellular calcium (Ca^2+^)_i_ was measured using the ratiometric Ca^2+^ probe Fura-2 with an IX81-ZDC inverted microscope (Olympus) controlled by Cell^R software (Olympus). MIN6 cells grown on poly-L lysine coated coverslips were equilibrated in glucose-free KRBH buffer and loaded with Fura-2-AM (Life Technologies) at 37°C with 5% CO_2_ for 30 min. All used compounds, alone or combined, were pre-incubated in the same conditions together with Fura-2-AM. After pre-incubation, the coverslips were mounted in a custom made perfusion chamber placed in an environmentally controlled culture chamber (OKO Lab) for perfusion. The cells were perfused for 2–3 min with glucose-free KRBH buffer (+/- testing compounds) for baseline detection followed by the effective stimulation (glucose +/- testing compounds). The imaging paradigm consisted of alternating excitation at 340 nm and 380 nm (300 ms each) and emission detection at 510 nm with a CCD camera (Hamamatsu). Images were taken every second and analyzed offline using Cell^R software. Individual cells were manually segmented and the 340/380 nm ratio of emission at 510 nm was calculated for each time point as indicative of (Ca^2+^)_i_ levels.

### The METSIM Study

A total of 20 fatty acids from the erythrocyte membranes were determined in 1,332 non-diabetic men from the METSIM (Metabolic Syndrome in Men) Study [8] (age 55±5 years, body mass index 26.4±3.5 kg/m^2^; mean ± SD) from fasting blood samples by gas chromatography, as previously described [9]. The concentrations of fatty acids were compared between participants with (N = 182) and without (N = 1,150) statin treatment by the t-test. Given 23 tests performed, p<0.002 was considered statistically significant. All participants gave a written informed consent. The study and the consent were approved by the Ethics Committee of the University of Kuopio and Kuopio University Hospital, and carried out in accordance with the Helsinki Declaration.

### Statistical analysis

Data (presented as mean±SEM relative to control) were analyzed with Mann-Whitney test or t-test, p<0.05 was considered statistically significant.

## Results

### Simvastatin but not pravastatin decreases insulin secretion

**Fig 1:**
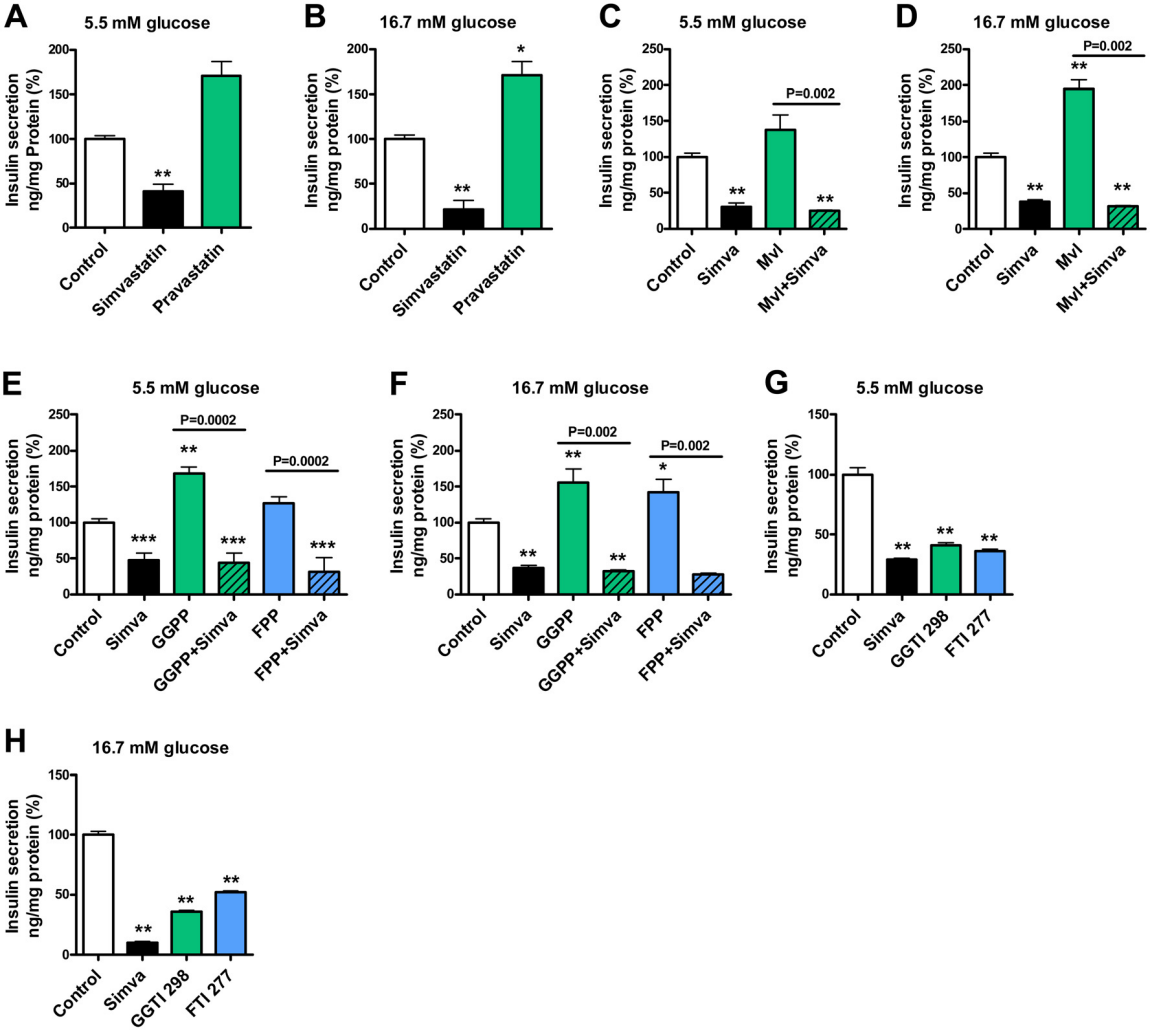
Effect of simvastatin, pravastatin and modulators of cholesterol biosynthesis pathway on glucose-stimulated insulin secretion in MIN6 β-cells. The effects of simvastatin (Simva, 14.3 μM) and pravastatin (26.3 μM) on insulin secretion at 5.5 mM (**A**) and 16.7 mM glucose concentrations (**B**); the effect of DL-mevalolactone (Mvl, 1 mM) on insulin secretion at 5.5 mM (**C**) and 16.7 mM glucose concentrations; the effect of the activation of isoprenoid intermediates geranylgeranyl pyrophosphate (GGPP) and farnesyl pyrophosphate (FPP) with geranylgeranyl pyrophosphate ammonium salt (GGPP, 20 μM) and farnesyl pyrophosphate ammonium salt (FPP, 20 μM) on insulin secretion at 5.5 mM (**E**) and 16.7 mM glucose concentrations (**F**); the effect of the inhibition of isoprenoid intermediates GGPP and FPP with geranylgeranyltransferase inhibitor (GGTI-298, 20 μM) and farnesyltransferase inhibitor (FTI-277, 20 μM) on insulin secretion at 5.5 mM (**G**) and 16.7 mM glucose concentrations (**H**); and the effect of the Rho/Rac/CDC42 activators (R/R/C actv) at 1, 2.5 and 5 μg/ml alone (**I**) or in combination with simvastatin (**J**) on insulin secretion at 16.7 mM glucose concentration. Insulin secretion was normalized with protein concentration, data are mean ± SEM relative to control (100%). p values were calculated with the Mann-Whitney test, *p<0.05, **p<0.01, ***p<0.001 compared to control, ^##^p<0.01, ^###^p<0.001 compared to simvastatin treatment. Each group had 6 replicates, except for I and J which had 8 replicates.

We treated MIN6 β-cells with 14.3 μM simvastatin (as in all simvastatin experiments, unless otherwise stated) and 26.3 μM pravastatin (as in all pravastatin experiments) at normal (5.5 mM) and high glucose concentrations (16.7 mM). At 5.5 mM glucose (Fig 1A) simvastatin decreased insulin secretion by 59% compared to control (p<0.01), whereas treatment with pravastatin increased insulin secretion nonsignificantly by 71% compared to control. At 16.7 mM glucose (Fig 1B) simvastatin decreased insulin secretion by 79%, and pravastatin increased insulin secretion by 71% compared to control (p<0.01 and p<0.05).

### Effect of simvastatin on insulin secretion in the cholesterol biosynthesis pathway

We treated MIN6 β-cells with simvastatin and 1 mM of DL-mevalolactone, an activator of mevalonate synthesis (intermediate in cholesterol biosynthesis). At 5.5 mM glucose concentration (Fig 1C) treatment of MIN6 β-cells with simvastatin decreased insulin secretion by 69% whereas treatment with DL-mevalolactone non-significantly increased insulin secretion by 38%, and treatment with both simvastatin and DL-mevalolactone decreased insulin secretion by 75% compared to control (p<0.01). When compared to DL-mevalolactone alone, treatment with both simvastatin and DL-mevalolactone decreased insulin secretion significantly (p = 0.002). At 16.7 mM glucose (Fig 1D) simvastatin decreased insulin secretion by 62%, whereas DL-mevalolactone increased insulin secretion by 95%, and treatment with both simvastatin and DL-mevalolactone decreased insulin secretion by 68% compared to control (p<0.01). When compared to DL-mevalolactone alone, treatment with both simvastatin and DL-mevalolactone decreased insulin secretion (p<0.002).

We treated MIN6 β-cells with simvastatin and activators of isoprenoid intermediates geranylgeranyl pyrophosphate (GGPP) and farnesyl pyrophosphate (FPP) (both 20 μM). At 5.5 mM glucose (Fig 1E) treatment with simvastatin decreased insulin secretion by 53%, whereas treatment with GGPP activator increased insulin secretion by 68% (p<0.01), and treatment with FPP activator increased insulin secretion nonsignificantly by 27%. Treatment with both simvastatin and GGPP activator decreased insulin secretion by 56%, compared to control (p<0.001) and GGPP activator alone (p = 0.0002). Treatment with both simvastatin and FPP activator decreased insulin secretion by 69%, compared to control (p<0.001) and FPP activator alone (p = 0.0002). At 16.7 mM glucose (Fig 1F) treatment with simvastatin decreased insulin secretion by 63%, whereas treatment with GGPP activator increased insulin secretion by 56%, and treatment with FPP activator increased insulin secretion by 42% (p<0.05). Treatment with both simvastatin and GGPP activator decreased insulin secretion by 68%, compared to control (p<0.01) and GGPP activator alone (p = 0.002). Treatment with both simvastatin and FPP activator decreased insulin secretion by 72%, compared to control (p<0.01), and FPP activator treatment alone (p = 0.002).

We treated MIN6 β-cells with simvastatin and inhibitors (20 μM) of geranylgeranyl pyrophosphate (GGTI-298) and farnesyl pyrophosphate (FTI-277). Simvastatin treatment at 5.5 mM glucose (Fig 1G) decreased insulin secretion by 71%, treatment with GGTI-298 by 59%, and treatment with FTI-277 by 64%, compared to control (p<0.01). At 16.7 mM glucose (Fig 1H) simvastatin decreased insulin secretion by 90%, treatment with GGTI-298 by 64%, and treatment with FTI-277 by 48% compared to control (p<0.01).

### Simvastatin impairs tolbutamide-, KCl-, nifedipine-, acetylcholine- and PMA-mediated insulin secretion

**Fig 2:**
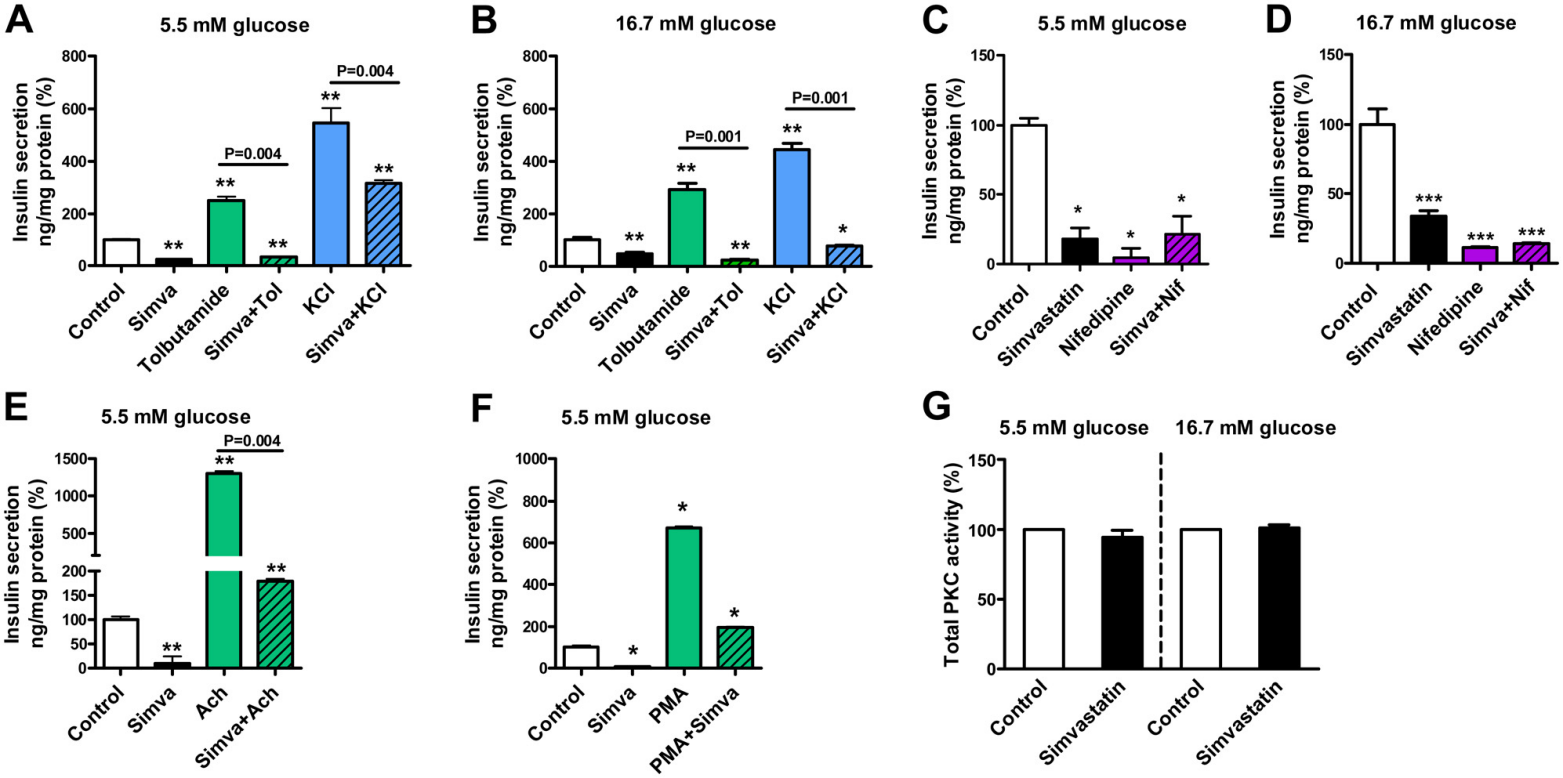
Effect of simvastatin on insulin secretion mediated by ATP dependent potassium channels, L-type voltage-gated calcium channels, acetylcholine, and phorbol myristate acetate in MIN6 β-cells. The effect of simvastatin (Simva, 14.3 μM) on insulin secretion stimulated by tolbutamide (Tol, 100 μM) and potassium chloride (KCl, 40 mM) at 5.5 mM (**A**) and 16.7 mM glucose concentrations (**B**); the effect of simvastatin and nifedipine (Nif, 5 μM) on insulin secretion at 5.5 mM (**C**) and 16.7 mM (**D**) glucose concentrations; the effect of simvastatin on insulin secretion stimulated by acetylcholine (Ach, 10 μM) at 5.5 mM glucose concentration (**E**); and the effect of simvastatin on insulin secretion stimulated by phorbol myristate acetate (PMA, 0.5 μM) at 5.5 mM glucose concentration (**F**); and the effect of simvastatin (14.3 μM) on the PKC activity in MIN6 β-cells at 5.5 and 16.7 mM glucose concentration (**G**). Insulin secretion was normalized with protein concentration, data are mean ± SEM relative to control (100%). p values were calculated with the Mann-Whitney test, *p<0.05, **p<0.01 compared to control. The number of replicates was 6 for A and E, 8 for B, C, and D, 4 for F, and 2–4 for G.

We treated MIN6 β-cells with simvastatin, 100 μM tolbutamide and 40 mM potassium chloride (KCl). At 5.5 mM glucose (Fig 2A) simvastatin decreased insulin secretion by 74%, whereas tolbutamide treatment increased insulin secretion by 150%, and treatment with KCl increased insulin secretion by 444% compared to control (p<0.01). Simvastatin decreased both tolbutamide- and KCl-induced insulin secretion (p = 0.004) to 33 and 316% of control. At 16.7 mM glucose (Fig 2B) simvastatin decreased insulin secretion by 51%, whereas treatment with tolbutamide increased insulin secretion by 192%, and KCl treatment increased insulin secretion by 345% (p<0.01). Simvastatin decreased both tolbutamide- and KCl-induced insulin secretion (p = 0.001) to 33 and 75% of control.

We treated MIN6 β-cells with simvastatin and 5 μM nifedipine. At 5.5 mM glucose (Fig 2C) simvastatin decreased insulin secretion by 82% (p<0.05), nifedipine by 96% (p<0.05), and a combination of simvastatin and nifedipine by 78% (p<0.05). At 16.7mM glucose (Fig 2D) simvastatin decreased insulin secretion by 66%, nifedipine by 89%, and a combination of simvastatin and nifedipine by 85% (p<0.001).

We treated MIN6 β-cells with simvastatin and 10 μM acetylcholine at 5.5 mM glucose (Fig 2E). Simvastatin decreased insulin secretion by 90%, whereas acetylcholine increased insulin secretion by 1194% (p<0.01). Simvastatin decreased acetylcholine-induced insulin secretion (p = 0.004) to 179% of control.

We treated MIN6 β-cells with simvastatin and 0.5 μM phorbol myristate acetate (PMA), an activator of protein kinase C (PKC) (Fig 2F) at 5.5 mM glucose. Simvastatin decreased insulin secretion by 93%, whereas PMA increased insulin secretion by 572% (p<0.05). Simvastatin decreased PMA-induced insulin secretion (p<0.05) to 195% of control. Simvastatin (14.3 μM) had no significant effect on PKC activity in MIN6 β-cells at 5.5 and 16.7 mM concentrations (Fig 2G).

### Simvastatin impairs insulin secretion mediated by the GPR40 signaling pathway

**Fig 3:**
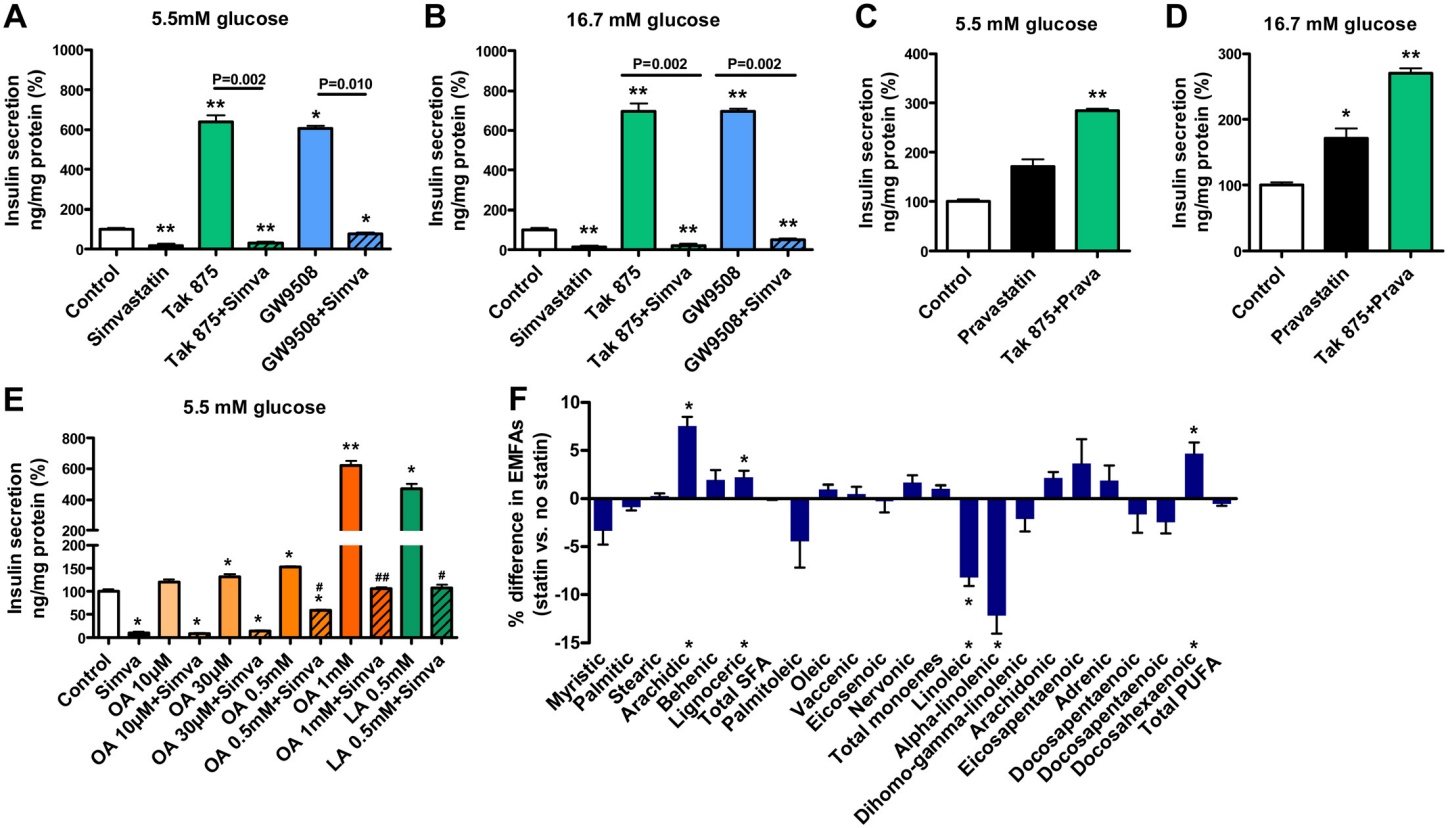
Effect of simvastatin on the GPR40 signaling pathway in MIN6 β-cells and on the erythrocyte membrane fatty acid (EMFA) levels in humans. The effect of simvastatin (Simva, 14.3 μM) on insulin secretion stimulated by GPR40 agonists TAK875 (40 μM) and GW9508 (40 μM) at 5.5 mM (**A**) and 16.7 mM glucose concentrations (**B**); the effect of pravastatin (26.3 μM) alone or in combination with TAK875 on insulin secretion at 5.5 mM (**C**) and 16.7 mM glucose concentrations (**D**); the effect of oleic acid (OA) at 10 μM, 30 μM, 0.5 mM and 1 mM concentrations and linoleic acid (LA) 0.5 mM concentration alone or in combination with simvastatin on insulin secretion at 5.5 mM glucose concentration (**E**); the effect of simvastatin treatment on the levels of EMFAs in 1,332 non-diabetic men from the METSIM study (**F**); SFA—total saturated fatty acids, PUFA—total polyunsaturated fatty acids. Insulin secretion were normalized with protein concentration, data are mean ± SEM relative to control (100%). p values were calculated with the Mann-Whitney test (A-E) or with t-test (F), *p<0.05, **p<0.01 compared to control, p^#^<0.05, p^##^<0.01 compared to simvastatin treatment. Each group has 6 (4–6 in E) replicates.

We treated MIN6 β-cells with simvastatin and GPR40 agonists (40 μM) TAK875 and GW9508. At 5.5 mM glucose concentration (Fig 3A) simvastatin decreased insulin secretion by 82%, whereas treatment with TAK875 increased insulin secretion by 538% and treatment with GW9508 by 596% (p<0.05). Simvastatin decreased TAK875- and GW9508-induced insulin secretion (p = 0.002 and 0.010, respectively) to 33 and 77% of control. At 16.7 mM glucose (Fig 3B) simvastatin treatment decreased insulin secretion by 85%, whereas treatment with TAK875 increased insulin secretion by 596%, and treatment with GW9508 increased insulin secretion by 598% (p<0.01). Simvastatin decreased TAK875- and GW9508-induced insulin secretion (p = 0.002) to 22 and 52% of control.

To compare the effects of simvastatin and pravastatin we treated MIN6 β-cells with pravastatin and 40 μM TAK875. At 5.5 mM glucose (Fig 3C) pravastatin increased insulin secretion by 71% (p = 0.055), and treatment with both pravastatin and TAK875 increased insulin secretion by 185% compared to control (p<0.01). At 16.7 mM glucose (Fig 3D) pravastatin increased insulin secretion by 71% (p = 0.025) and treatment with pravastatin and TAK875 increased insulin secretion by 171% compared to control (p<0.01).

To investigate the role of fatty acid concentrations on the GPR40 signaling pathway, we treated MIN6 β-cells with simvastatin, oleic acid (OA) at 10 and 30 μM, 0.5 and 1 mM concentrations and 0.5 mM linoleic acid (LA) at 5.5 mM glucose (Fig 3E). Simvastatin decreased insulin secretion by 89% (p<0.05) compared to control. Treatment with OA increased insulin secretion in a dose-dependent manner by 21% (10 μM), and significantly (p<0.05) by 31% (30 μM), 54% (0.5 mM) and 521% (1 mM). LA (0.5 mM) increased insulin secretion by 372% (p<0.05). Simvastatin decreased OA- and LA-induced insulin secretion at all concentrations, but OA at ≥0.5 mM concentration and LA at 0.5 mM concentration partially restored insulin secretion (p<0.05 compared to simvastatin treatment alone).

We examined the levels of 20 erythrocyte membrane fatty acids in 1,332 non-diabetic men from the METSIM Study (Fig 3F). The levels of linoleic and alpha-linoleic acids were significantly (p<0.002) lower, whereas the levels of arachidic, lignoceric and docosapentaenoic acids were significantly higher in men receiving statins compared with non-users of statins.

### Simvastatin impairs GLP-1 receptor-mediated insulin secretion but not GPR119-mediated insulin secretion

**Fig 4:**
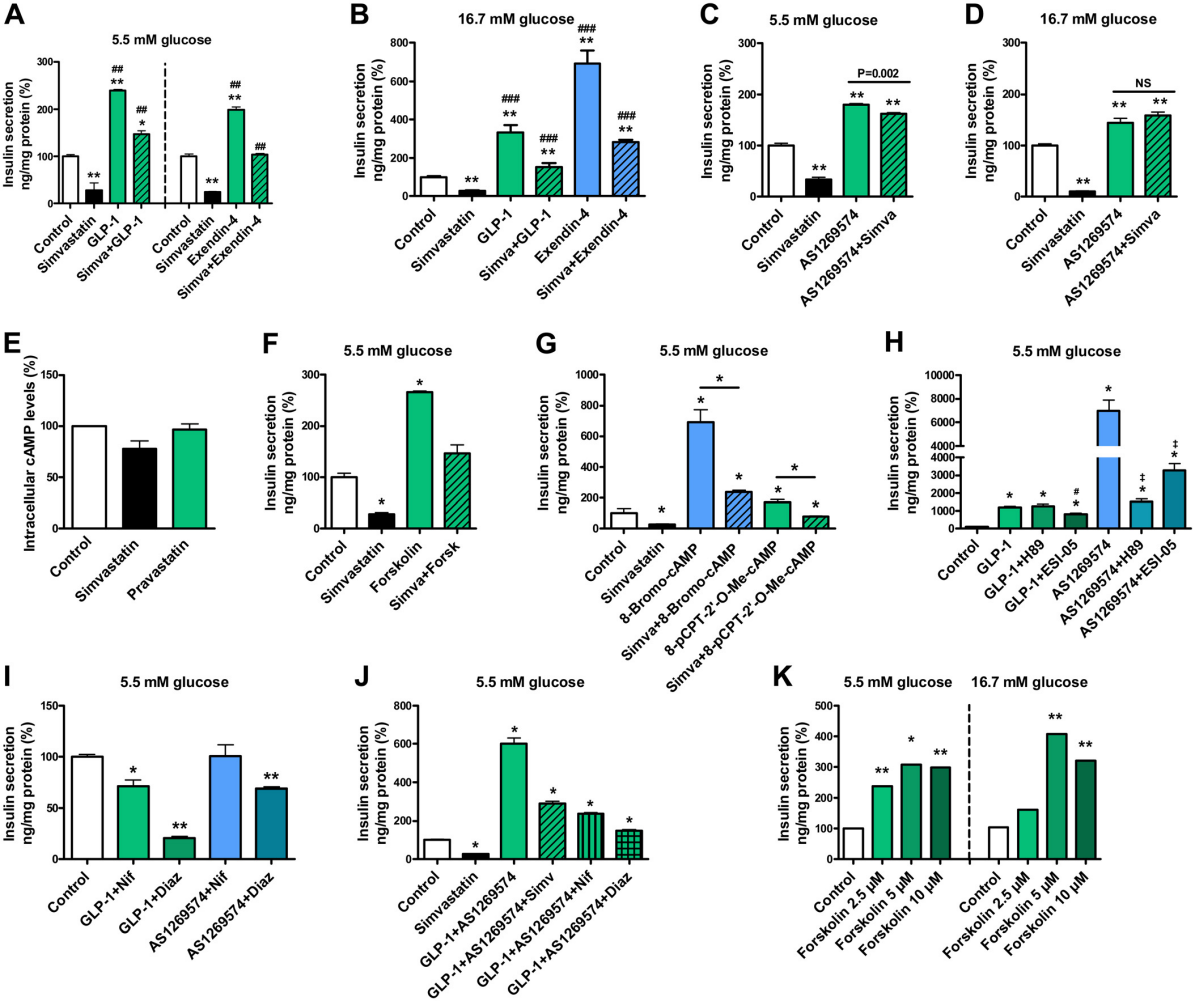
Effect of simvastatin on the glucagon-like peptide 1 (GLP-1) receptor and GPR119 pathways in MIN6 β-cells and GPR119 cell line. The effect GLP-1 amide (100 nM) and a GLP-1 agonist exendin-4 (20 nM) alone or in combination with simvastatin (Simva) on insulin secretion at 5.5 mM (**A**) and 16.7 mM glucose concentrations (**B**); the effect of the GPR119 agonist AS-1269574 (40 μM) alone or in combination with simvastatin on insulin secretion at 5.5 mM (**C**) and 16.7 mM glucose concentrations (**D**); the effect of simvastatin and pravastatin (26.3 μM) on cAMP concentration in GPR119 receptor overexpressing CHO-K1 cell lines using cAMP HTRF^™^ functional assay (**E**); the effect of the cAMP activator forskolin (Forsk, 10 μM) alone or in combination with simvastatin on insulin secretion at 5.5 mM glucose concentration (**F**); the effect of simvastatin on insulin secretion stimulated by a cAMP analog that activates both protein kinase A (PKA) and Epac2 (8-Bromo-cAMP, 1 mM), and a cAMP analog specific for activation of Epacs (8-pCPT-2′-O-Me-cAMP, 50μM) (**G**); the effect of GLP-1 amide (100 nM) and AS-1269574 (40 μM) alone or in combination with PKA specific inhibitor H89 (10 μM) or Epac specific inhibitor ESI-05 (20 μM) on insulin secretion at 5.5 mM glucose concentration (**H**); the effect of GLP-1 amide (100 nM) and AS-1269574 (40 μM) in combination with nifedipine (Nif, 5 μM) or diazoxide (Diaz, 250 μM) on insulin secretion at 5.5 mM glucose concentration (**I**); the effect of a combination of GLP-1 amide (100 nM) and AS-1269574 (40 μM) alone or with simvastatin, nifedipine or diazoxide on insulin secretion at 5.5 mM glucose concentration (**J**); the effect of forskolin at 3 different concentrations (2.5, 5, and 10 μM) on insulin secretion in calcium-free KRBH buffer at 5.5 and 16.7 mM glucose concentration (**K**). Insulin secretion was normalized with protein concentration, data are mean ± SEM relative to control (100%). Simvastatin was used in concentration of 14.3 μM. p values were calculated with the Mann-Whitney test, *p<0.05, **p<0.01 compared to control or other reference as indicated, p^##^<0.01 and p^###^<0.001 compared to simvastatin treatment. Each group had 8 (A, B), 6 (C, D, I, K), or 4 (F, G, H, J) replicates. See also S1 Fig.

We treated MIN6 β-cells with simvastatin, 100 nM glucagon like peptide-1 (GLP-1) amide and 20 nM GLP-1 receptor agonist exendin-4. At 5.5 mM glucose (Fig 4A) simvastatin decreased insulin secretion by 73–76% (p<0.01), whereas GLP-1 increased insulin secretion by 139% and exendin-4 increased insulin secretion by 99% (p<0.01). Simvastatin decreased GLP-1- and exendin-4-stimulated insulin secretion to 147% and 104% of control, indicating that GLP-1 and exendin-4 can partially restore simvastatin-induced defect in insulin secretion (p<0.01 compared to simvastatin). Similarly, at 16.7 mM glucose (Fig 4B) simvastatin decreased insulin secretion by 73%, whereas GLP-1 increased insulin secretion by 233%, and exendin-4 by 591% (p<0.01). Simvastatin decreased GLP-1- and exendin-4-stimulated insulin secretion to 153 and 282% of control, indicating that GLP-1 and exendin-4 can partially restore simvastatin-induced defect in insulin secretion (p<0.001 compared to simvastatin).

We treated MIN6 β-cells with simvastatin and a GPR119 agonist AS-1269574 (40 μM). At 5.5 mM glucose (Fig 4C) simvastatin decreased insulin secretion by 66% whereas treatment with AS-1269574 increased insulin secretion by 80% (p<0.01). Simvastatin only slightly decreased AS-1269574-stimulated insulin secretion (p = 0.002, 162% of control), suggesting that a GPR119 agonist AS-1269574 was able to restore simvastatin-induced defect in insulin secretion. At 16.7 mM glucose (Fig 4D) simvastatin decreased insulin secretion by 90%, whereas AS-1269574 increased insulin secretion by 44%. Simvastatin had no significant effect on AS-1269574-stimulated insulin secretion, and AS-1269574 completely restored simvastatin-induced defect in insulin secretion.

### Simvastatin and intracellular cAMP levels

GPR119-overexpressing CHO-K1 cells were treated with simvastatin and pravastatin to assess cAMP concentration inside the cell using cAMP HTRF^™^ functional assay in antagonist mode (Fig 4E). The cells were first treated with simvastatin or pravastatin for 30 minutes, and then stimulated with GPR119 agonist oleoylethanolamide, which increases cAMP levels. Simvastatin decreased cAMP concentration nonsignificantly by 22% and pravastatin had a negligible effect (-3%) on cAMP concentration. In MIN6 cells, membrane-permeable cAMP activator forskolin (10 μM) increased insulin secretion by 166% (p<0.05) and simvastatin decreased forskolin-stimulated insulin secretion to 146% of control, suggesting that cAMP activation can restore simvastatin-induced decrease in insulin secretion (Fig 4F).

### Simvastatin decreases PKA- and Epac2-mediated insulin secretion

We treated MIN6 β-cells with simvastatin, with 1 mM 8-bromo-cAMP, which activates both protein kinase A (PKA) and exchange protein activated by cAMP 2 (Epac2), and with 50 μM 8-pCPT-2′-O-Me-cAMP, a cAMP analog specific for activation of Epacs. At 5.5 mM glucose simvastatin decreased insulin secretion by 75%, whereas 8-bromo-cAMP increased insulin secretion by 593%, and 8-pCPT-2′-O-Me-cAMP by 69% (p<0.05). Simvastatin decreased both 8-bromo-cAMP- and 8-pCPT-2′-O-Me-cAMP-induced insulin secretion (p<0.05) (Fig 4G).

To examine the role of PKA and Epac2 in GLP-1 receptor and GPR119-stimulated insulin secretion we treated MIN6 β-cells with a specific inhibitor of PKA, H89 (10 μM), and a specific inhibitor of Epac2, ESI-05 (20 μM). PKA inhibition by H89 decreased only AS-1269574-stimulated insulin secretion (GPR119 pathway), whereas Epac2 inhibition by ESI-05 decreased both GLP-1- and AS-1269574-stimulated insulin secretion (p<0.05) (Fig 4H). At 16.7 mM glucose simvastatin had no significant effect on protein expression of GLP-1 receptor (GLP-1R) (Figure A in S1 Fig), PKA alpha catalytic subunit (Figure B in S1 Fig), PKA beta regulatory subunit (Figure C in S1 Fig) or Epac2 (Figure D in S1 Fig).

### Voltage-gated calcium channels and K_ATP_ channels in GLP-1 receptor and GPR119 mediated insulin secretion

To examine the role of VGCCs and K_ATP_ channels in GLP-1- and GPR119-mediated insulin secretion we treated MIN6 β-cells with a combination of GLP-1 amide and nifedipine (5 μM) or diazoxide (250 μM), and a combination of AS-1269574 and nifedipine or diazoxide (Fig 4I). AS-1269574 better restored nifedipine- or diazoxide-decreased insulin secretion (to 101 and 69% of control) than GLP-1 amide (to 71 and 21% of control). A combination treatment with GLP-1 amide and AS-1269574 increased insulin secretion by 502%, and addition of simvastatin, nifedipine or diazoxide decreased GLP-1- and AS-1269574-stimulated insulin secretion to the level of 291, 238 and 147% of control (p<0.05) (Fig 4J). Adenylate cyclase activator forskolin (at 2.5 μM, 5 μM, and 10 μM concentration) increased insulin secretion significantly (p<0.05) in Ca^2+^-free KRBH buffer at both 5.5 mM (by 138, 209, and 198%) and 16.7 mM glucose (by 308 and 222% at two higher concentrations) (Fig 4K), suggesting Ca^2+^-independent effects of cAMP activation on insulin secretion.

### Effect of simvastatin on the intracellular Ca^2+^ levels

**Fig 5:**
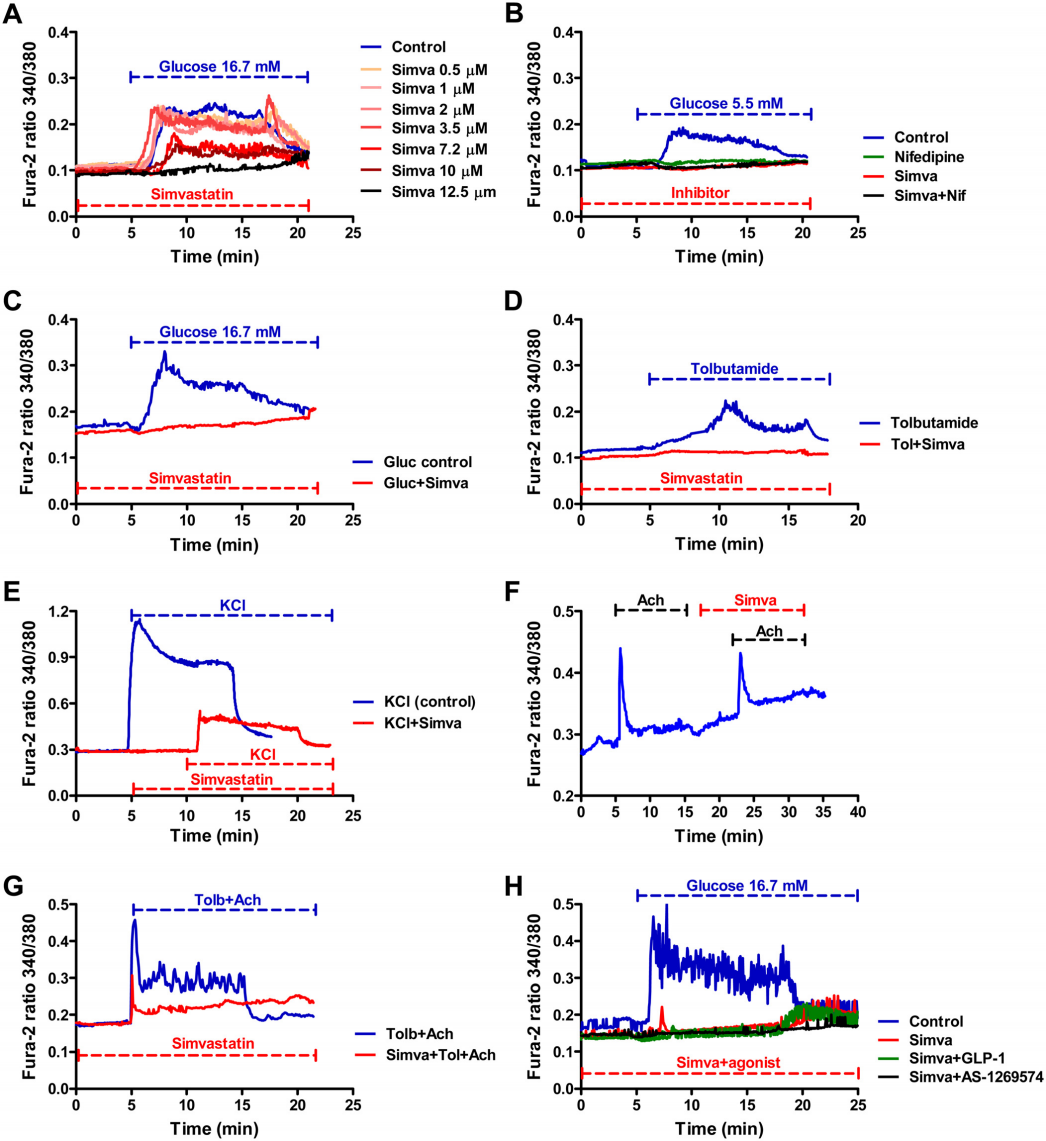
Effect of simvastatin (Simva) and other compounds on the intracellular calcium levels in MIN6 β-cells. The effect of simvastatin (0.5–12.5 μM) in the presence of 16.7 mM glucose (**A**); effect of simvastatin (14.4 μM) and nifedipine (Nif, 5 μM) in the presence of 5.5 mM glucose (**B**); effect of simvastatin (14.4 μM) in the presence of 16.7 mM glucose (**C**); effect of tolbutamide (Tol, 100 μM) alone or in combination with simvastatin (14.4 μM) (**D**); effect of KCl (40 mM) alone or in combination with simvastatin (7.2 μM) (**E**); effect of acetylcholine (Ach, 10 μM) alone or in combination with simvastatin (7.2 μM) (**F**); effect of a combination of tolbutamide and acetylcholine with or without simvastatin (14.4 μM) (**G**); effect of simvastatin (14.3 μM) alone or in combination with either GLP-1 (100 nM) or GPR119 agonist AS-1269574 (40 μM) in the presence of 16.7 mM glucose (**H**). Dashed lines represent the duration of the treatments.

Simvastatin at 8 different concentrations (0.5–12.5 μM in Fig 5A and 14.4 μM in Fig 5C) dose-dependently inhibited the increase in (Ca^2+^)_i_ levels stimulated by 16.7 mM glucose. In response to glucose (5.5 mM), simvastatin (14.4 μM) and nifedipine (5 μM) completely and independently blocked Ca^2+^ influx (Fig 5B). To further characterize the Ca^2+^ influx-blocker effects of simvastatin, we stimulated MIN6 cells with tolbutamide and acetylcholine alone or in combination. Simvastatin abolished the (Ca^2+^)_i_ increase stimulated by tolbutamide (Fig 5D). Furthermore, simvastatin (7.2 μM) inhibited an increase in the (Ca^2+^)_i_ stimulated by KCl (Fig 5E) which directly depolarizes membrane. Simvastatin failed to effectively inhibit the acetylcholine-stimulated increase in (Ca^2+^)_i_ (Fig 5F), in contrast to the complete inhibition found for glucose and tolbutamide. Similarly, simvastatin only partially inhibited the Ca^2+^ response under stimulation of tolbutamide and acetylcholine confirming that simvastatin has a minor or no effect on acetylcholine-stimulated Ca^2+^ release from the endoplasmic reticulum. (Fig 5G). Treatments with GLP-1 or AS-1269574 were not able to restore the inhibitory effect of simvastatin on (Ca^2+^)_i_ levels stimulated by 16.7 mM glucose (Fig 5H).

### Simvastatin decreases Ca2+ release from endoplasmic reticulum

**Fig 6:**
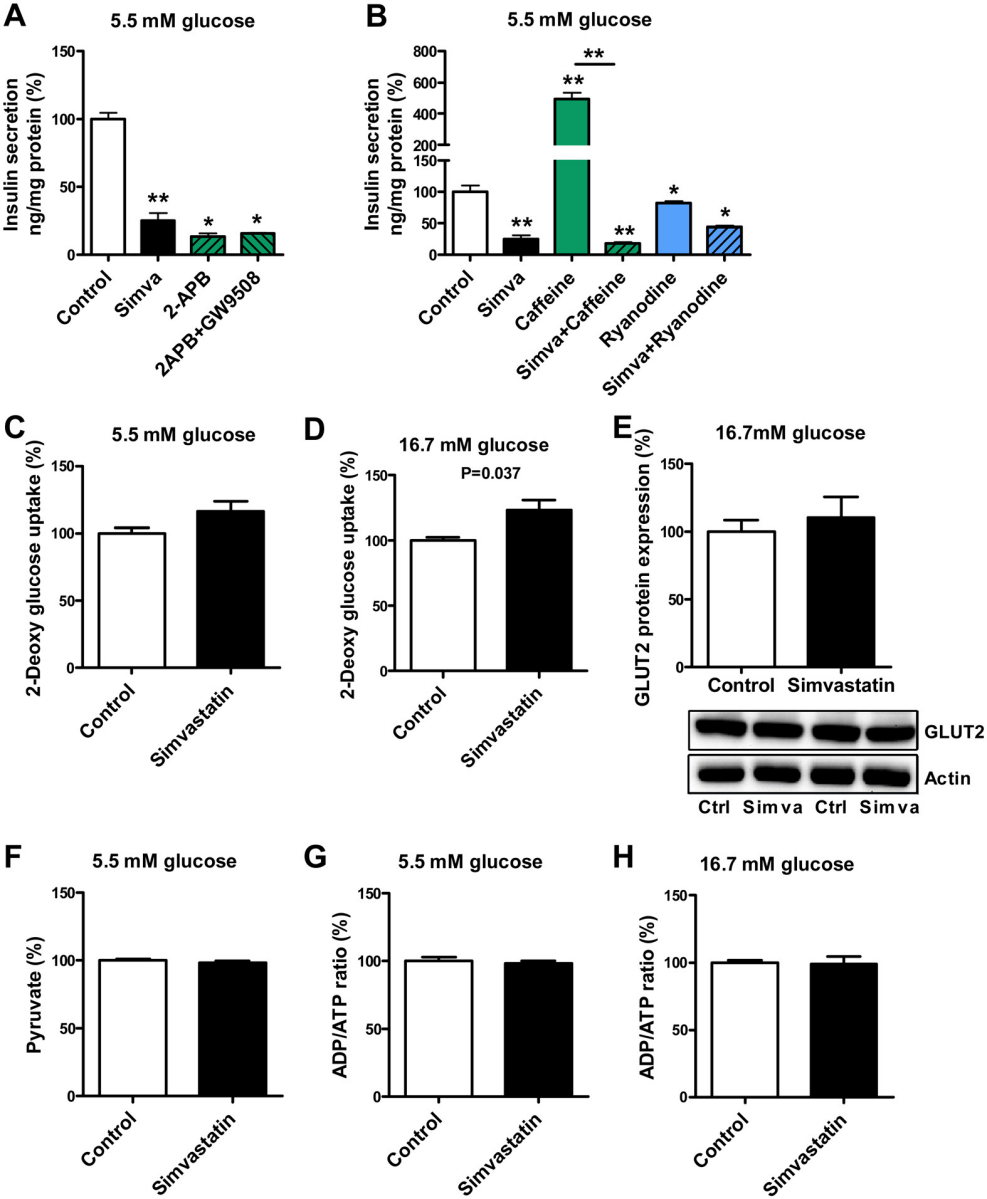
Effect of simvastatin on intracellular calcium and the glycolysis pathway in MIN6 β-cells. The effect of the 2-aminoethoxydiphenylborate (2-APB, 100 μM), a functional and membrane permeable IP3 receptor antagonist, on insulin secretion, and the effect of 2-APB on insulin secretion stimulated by GPR40 activator GW9580 (40 μM) in MIN6 β-cells (**A**); the effect of simvastatin on insulin secretion stimulated by the ryanodine receptor activator caffeine (10 mM) and the effect of ryanodine receptor inhibitor ryanodine (5 μM) on insulin secretion at 5.5 mM glucose concentration (**B**); the effect of simvastatin on glucose uptake at 5.5 mM (**C**) and 16.7 mM glucose concentrations (**D**), GLUT2 protein expression and corresponding western blots at 16.7 mM glucose concentrations (**E**), pyruvate levels at 5.5 mM glucose concentration (**F**), and the ADP/ATP ratio at 5.5 mM (**G**) and 16.7 mM glucose concentrations (**H**) in MIN6 β-cells. Insulin secretion values were normalized with protein concentration. GLUT2 protein expression values were normalized with actin protein levels. Data are mean ± SEM relative to control (Ctrl) (100%). p values were calculated with the Mann-Whitney test, *p<0.05, **p<0.01 compared to control. Each group has 6 (A-D, F-H) or 8 (E) replicates.

To investigate the role of Ca^2+^ release from endoplasmic reticulum we used modulators of endoplasmic reticulum receptors in MIN6 β-cells (Fig 6A and 6B). At 5.5 mM glucose 100 μM 2-aminoethoxydiphenylborate (2-APB), an antagonist of inositol triphosphate (IP3 receptors, decreased insulin secretion by 86% compared to control (p<0.05), and also decreased insulin secretion stimulated by GPR40 agonist GW9580 to 16% of control (Fig 6A). At 5.5 mM glucose simvastatin decreased insulin secretion by 75% whereas 10 mM caffeine, an activator of ryanodine receptors (RyR), increased insulin secretion by 394% (p<0.01). Simvastatin almost completely abolished caffeine-stimulated insulin secretion (18% of control, p<0.01). 5 μM of ryanodine, a RyR inhibitor, decreased insulin secretion by 18%, and a combination of ryanodine with simvastatin by 57% compared to control (p<0.05) (Fig 6B).

### Effects of simvastatin on glucose uptake, glycolysis and insulin signaling pathways

MIN6 β-cells were treated with simvastatin for 1 hour and glucose uptake was measured using the glucose analogue 2-deoxy-[1,2-^3^H]-glucose. Simvastatin had no significant effect on glucose uptake at 5.5 mM glucose concentration (Fig 6C), whereas at 16.7 mM glucose simvastatin treatment slightly increased glucose uptake compared to control (p = 0.037) (Fig 6D). Simvastatin had no significant effect on GLUT2 protein expression at 16.7 mM glucose (Fig 6E), pyruvate levels at 5.5 mM glucose (Fig 6F) or on the ADP/ATP ratio at 5.5 mM (Fig 6G) and 16.7 mM glucose (Fig 6H).

Treatment of MIN6 β-cells with simvastatin had no significant effect on protein expression of insulin receptor (Figure A-B in S2 Fig), phosphorylation of insulin receptor at 5.5 mM glucose (Figure C in S2 Fig), protein expression of insulin receptor substrate-1 (IRS1) (Figure E-F in S2 Fig), or on insulin receptor substrate-2 (IRS2) (Figure G-H in S2 Fig). However, simvastatin decreased phosphorylation of insulin receptor at 16.7 mM glucose (p = 0.006) (Figure D in S2 Fig) and serine phosphorylation of IRS1 at both 5.5 mM (p = 0.05) and 16.7 mM (p = 0.004) glucose concentrations (Figure I-J in S2 Fig).

Simvastatin decreased AKT activation and phosphorylation significantly at both 5.5 mM (p = 0.004, Figure A in S3 Fig) and 16.7 mM glucose concentrations (p = 0.006, Figure B in S3 Fig), whereas pravastatin had no effect on phosphorylation and activation of AKT at 16.7 mM glucose (Figure C in S3 Fig). At 16.7 mM glucose inhibitors of isoprenoid intermediates GGTI-298 (20 μM) and FTI-277 (20 μM) decreased AKT activation and phosphorylation in a magnitude comparable to the effect of simvastatin (Figure D in S3 Fig). Acetylcholine (10 μM) had no effect on AKT phosphorylation and activation, whereas combined treatment with acetylcholine and simvastatin decreased AKT phosphorylation and activation significantly compared to control (p = 0.001) (Figure E in S3 Fig). GLP-1 at different concentrations (25–100 nM) was able to restore simvastatin-induced decrease in AKT phosphorylation and activation (p<0.05 compared to simvastatin treatment) (Figure F in S3 Fig). Two AKT inhibitors, MK2206 (1 μM) and perifosine (20 μM) increased insulin secretion (p<0.05), which argues against the importance of AKT inhibition in simvastatin-stimulated decrease in insulin secretion (Figure G in S3 Fig).

## Discussion

Impaired insulin secretion and insulin resistance are needed for the development of diabetes. Statins have been shown to inhibit insulin secretion from the pancreatic β-cells [5], and increase insulin resistance *via* NLRP3/caspase-1-mediated mechanisms in adipose tissue [10]. Recently we reported that both decreased insulin secretion and increased insulin resistance were associated with the development of type 2 diabetes in participants of the METSIM study [11].

**Fig 7:**
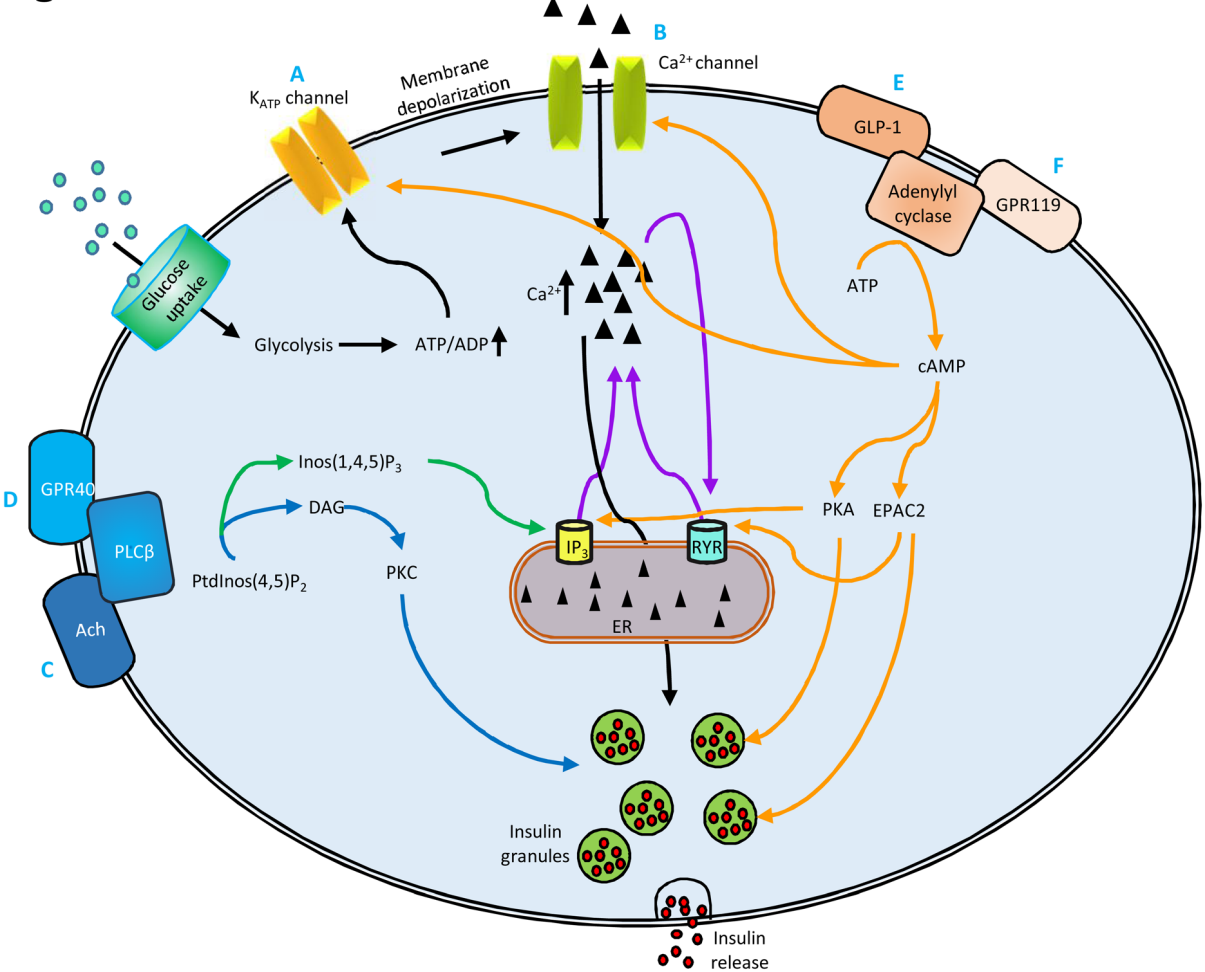
Different pathways of insulin secretion, links between them and effects of simvastatin on these pathways. Simvastatin (Simva) decreases insulin secretion mediated by ATP-sensitive potassium (K_ATP_) channels (A), membrane depolarization and voltage-gated calcium channels (B), and acetylcholine and GPR40 receptors (C and D). These effects may be attributable to simvastatin´s actions on K_ATP_ channels, calcium channels, or Ca^2+^ release from endoplasmic reticulum *via* inositol 3-phophate (IP3) and ryanodine receptors (RYR). Activation of GLP-1R (E) and GPR119 (F) pathway restores simvastatin-decreased insulin secretion. DAG—diacylglycerol, EPAC2—exchange protein activated by cAMP 2, ER—endoplasmic reticulum, Inos(1,4,5)P_3_ –inositol 1,4,5-triphosphate, PKA—protein kinase A, PLCβ–phospholipase C beta, PtdInos(4,5)P_2—_phosphatidylinositol 4,5-bisphosphate.

In the present study we showed that simvastatin decreased glucose-stimulated insulin secretion in MIN6 β-cells at normal glucose concentration (5.5 mM) by multiple mechanisms, including inhibitory effects on the VGCCs, acetylcholine pathway, and the GPR40 pathway, whereas simvastatin-induced impairment in insulin secretion was substantially less in the GLP-1 receptor and GPR119 pathways (Fig 7). Our results remained essentially unchanged at high glucose concentration (16.7 mM) indicating that simvastatin’s effect on insulin secretion is not substantially affected by the presence of hyperglycemia. In contrast, pravastatin did not decrease insulin secretion. Our observation of the opposite effects of simvastatin and pravastatin on glucose-stimulated insulin secretion is in agreement with previous reports showing that simvastatin, but not pravastatin, was associated with an increased risk of incident diabetes [1]. As simvastatin is lipophilic and pravastatin hydrophilic, their opposite effects on insulin secretion have been hypothesized to stem from differences in lipophilicity [4]. However, rosuvastatin is hydrophilic but increases substantially the risk of diabetes [3], and therefore lipophilicity cannot explain the differences in the risk of statins to induce diabetes. Further studies are needed to investigate the mechanisms of diabetogenity of different statins.

Simvastatin inhibits the rate-limiting step of cholesterol biosynthesis (HMG-CoA reductase). It also prevents the synthesis of isoprenoid intermediates derived from mevalonate, such as GGPP and FPP, which are known to induce prenylation of numerous cellular proteins. Our results on the stimulatory effects of mevalonate, GGPP and FPP on insulin secretion are in agreement with previously published findings [12]. We were also able to confirm that GGPP and FPP inhibitors reduced insulin secretion [12, 13]. As mevalonate, GGPP or FPP did not affect simvastatin induced decrease in insulin secretion, it is apparent that simvastatin does not exert its effect on insulin secretion through the inhibition of the cholesterol biosynthetic pathway. This is further supported by the fact that pravastatin, which should also completely block the cholesterol biosynthetic pathway at the concentration used [14], did not inhibit insulin secretion.

Simvastatin inhibited insulin secretion stimulated by tolbutamide which closes the K_ATP_ channels, and insulin secretion stimulated by KCl which directly depolarizes plasma membrane and leads to the opening of VGCCs. Simvastatin also inhibited glucose-induced raise in (Ca^2+^)_i_ in MIN6 cells in a dose-dependent manner, as well as decreased tolbutamide- and KCl-induced raise in (Ca^2+^)_i_. These results are in accordance with previous studies showing that simvastatin blocks VGCCs [5, 6]. The effect of simvastatin was comparable to that of the VGCC blocker nifedipine in our study.

Using the two GPR40 agonists, GW9580 and TAK875 we demonstrated for the first time that simvastatin inhibited GPR40-mediated insulin secretion at both normal (5.5 mM) and high (16.7 mM) glucose concentrations. GPR40 (also known as a free fatty acid receptor 1) is highly expressed in pancreatic β-cells [15] and mediates fatty-acid-induced enhancement of insulin secretion during hyperglycemia [16]. GPR40 activation leads to an increase in cytosolic Ca^2+^ concentration, activation of phospholipase C (PLC), and an increase in cAMP concentration [17, 18]. Unlike specific GPR40 agonists GW9580 and TAK875, oleic acid and linoleic acid at high concentrations restored insulin secretion decreased by simvastatin to the level of control, probably by stimulating other signaling pathways crucial for insulin secretion, including *de novo* synthesis of diacylglycerol (DAG) and phospholipids [19]. In the METSIM study cohort, statin treatment was associated with lower levels of linoleic and α-linolenic acid in erythrocyte membranes, possibly contributing to the statin-induced decrease in insulin secretion. GPR40 mediated insulin secretion is dependent on Ca^2+^ influx into the β-cell [18], and Ca^2+^ release from intracellular Ca^2+^ stores, such as endoplasmic reticulum [16]. Accordingly, we observed that an IP3 receptor (IP3R) antagonist 2-APB abolished GW9580 stimulated insulin secretion.

Acetylcholine (muscarinic M3) receptors belong to the same family of G-protein coupled receptors as GPR40 [20]. Muscarinic M3 receptors are present on the plasma membrane of β-cells and activate insulin secretion by stimulating Ca^2+^ release from endoplasmic reticulum *via* IP3R, formation of DAG, which activates PKC, and by potentiating exocytosis of insulin [21–24]. Our novel finding was that simvastatin significantly reduced, but did not abolish, the stimulatory effect of acetylcholine on insulin secretion and (Ca^2+^)_i_ increase in β-cells.

The experiments with DAG analog PMA showed that also a direct stimulation of PKC activity partially reversed simvastatin induced decrease in insulin secretion. On the other hand, the activity of PKC was not affected by simvastatin in our study. Although insulin secretion pathways mediated by acetylcholine, GPR40 and PMA, share similar mechanisms, a decrease in PMA- and acetylcholine-stimulated insulin secretion by simvastatin was less extensive compared to GPR40-mediated insulin secretion.

Stimulation of insulin secretion through the GPR40 pathway depends on the influx of extracellular Ca^2+^, K_ATP_ channels and delayed rectifier K^+^ channels. Simvastatin can exert its inhibitory effects through several targets in these pathways [18, 25–28]. Our results indicate that the effects of simvastatin on acetylcholine-stimulated insulin secretion cannot be explained by the inhibition of the most established pathways of muscarinic M3 receptor signaling, IP3-mediated Ca^2+^ release or PKC. Possible effects of simvastatin on other downstream molecules of the acetylcholine pathway and also its effect on other pathways which influence the dynamics of insulin granular exocytosis cannot be excluded and will require further studies.

We also observed that simvastatin affected insulin secretion mediated by the GLP-1 receptor and GPR119 pathways. Both GLP-1 receptor and GPR119 agonists increase insulin secretion by stimulating adenylate cyclase which catalyzes the conversion of ATP to cAMP [29, 30]. Simvastatin only partially reduced insulin secretion stimulated by GLP-1, exendin-4 or GPR119 agonist AS-1269574 suggesting that the stimulation of GLP-1 receptor and especially the GPR119 pathways can largely restore impaired insulin secretion associated with simvastatin. Forskolin, a direct activator of cAMP also restored insulin secretion decreased by simvastatin. Additionally, we found that inhibitory effects of nifedipine (VGCC blocker) and diazoxide (K_ATP_ channel opener) were less on GPR119-stimulated insulin secretion than on GLP-1 receptor-stimulated insulin secretion. Since simvastatin affects both VGCC and K_ATP_ channels [5, 6], this may explain why GPR119 agonist was more potent in restoring the simvastatin-induced decrease in insulin secretion than GLP-1 or exendin-4.

The downstream targets of cAMP, PKA and Epac2 are crucial in GLP-1 receptor-mediated insulin secretion [31–33], in the opening of VGCCs, and the closure of K_ATP_ channels. Our experiments with Epac2 and PKA inhibitors suggest the role of PKA and Epac2 in GPR119-mediated insulin secretion. Using cAMP analogs activating both PKA and Epac2 (8-bromo-cAMP), or specific activator of the Epacs (8-pCPT-2′-*O*-Me-cAMP), we showed for the first time that simvastatin reduced insulin secretion *via* both Epac2 and PKA pathways.

GPR119 and GLP-1 receptor pathways require the coupling of glucose-induced Ca^2+^ influx through VGCCs to enhance insulin exocytosis [34]. GLP-1 receptor pathway participates in Ca^2+^ release where Ca^2+^ influx through Ca^2+^ channels facilitates the release of Ca^2+^ from intracellular stores, such as endoplasmic reticulum [35, 36] *via* IP3R and RyR [37, 38]. GRP119 likely exerts similar effects since a cAMP activator forskolin has been found to potentiate caffeine-induced Ca^2+^ spikes in cultured β-cells [39]. We also showed that simvastatin decreased Ca^2+^ release from the endoplasmic reticulum by abolishing insulin secretion stimulated by a RyR activator caffeine.

We demonstrated that GLP-1 receptor and GPR119 agonists were not able to counteract the inhibitory effect of simvastatin on (Ca^2+^)_i_ levels after glucose stimulation. This suggests that Ca^2+^-independent effects of cAMP on insulin secretion [40, 41] might be responsible for the restoration of simvastatin-induced decrease in insulin secretion by the activation of these receptors. Accordingly, the cAMP activator forskolin stimulated insulin secretion from MIN6 cells even in Ca^2+^-free conditions in our study. cAMP is known to stimulate both the first phase of insulin secretion, formed by the Ca^2+^ influx through VGCCs acting on a limited set of readily releasable secretory granules, and the second phase of insulin secretion which involves both readily releasable pool and reserve pool insulin granules and is dependent on the energy metabolism of the β-cell and on amplifying pathways, such as GLP-1 receptor pathway [42–45]. GLP-1 receptor modulates the second phase of insulin secretion in both PKA and Epac dependent manner [43–45]. cAMP also promotes insulin release by a direct interaction with the secretory machinery [41]. We observed that simvastatin decreased the phosphorylation of several proteins involved in the insulin signaling pathway, such as insulin receptor, insulin receptor substrate-1 and AKT (pAKT). This may indicate that statin treatment induced impairment in the insulin signaling pathway in the MIN6-cells which could decrease insulin secretion. AKT inhibitors MK2206 and perifosine did not decrease but rather increased insulin secretion, similarly to a previous report [46]. Therefore simvastatin´s effect on pAKT is likely to be secondary.

Based on our results, we hypothesize that GLP-1 receptor and GPR119 agonists may improve insulin secretion in individuals treated with simvastatin better than sulfonylureas, since simvastatin completely blocks tolbutamide-induced insulin secretion, whereas it had a substantially smaller effect on GLP-1 receptor- and GPR119-induced insulin secretion. However, clinical studies are needed to determine whether GLP-1 receptor or GPR119 agonists decrease hyperglycemia more effectively than other treatments in patients with type 2 diabetes on simvastatin therapy.

In summary, our study reports for the first time that simvastatin decreases insulin secretion in MIN6 β-cells *via* multiple pathways including acetylcholine receptor, GPR40, and inhibition of Ca^2+^ release from intracellular stores, in addition to the known effects on the VGCC channels. The activation of GPR119 or GLP-1 receptor signaling partially restored the simvastatin-induced impairment in insulin secretion.

## Supporting Information

S1 Appendix Supplemental methods. (DOCX)

S2 Appendix Full blot information for S1–S3 Figs. (DOCX)

S3 Appendix Raw data files. (XLSX)

S1 Table List of cells, reagents, materials, assays, antibodies and other equipment used for the experiments and their sources. (DOCX)

S1 Fig Effect of simvastatin on protein expression of different proteins involved in GLP-1 signaling pathway in MIN6 β-cells. (DOCX)

S2 Fig Effect of simvastatin on protein expression of different proteins involved in insulin signaling pathway in MIN6 β-cells. (DOCX)

S3 Fig Effect of simvastatin and other compounds on AKT activation and phosphorylation in MIN6 β-cells. (DOCX)
