# Supplementary material for: Simvastatin Impairs Insulin Secretion by Multiple Mechanisms in MIN6 Cells
Source: PLoS One. 2015 Nov 11;10(11):e0142902. doi: 10.1371/journal.pone.0142902 (PMC4641640; doi:10.1371/journal.pone.0142902)
Supplement: S1 Appendix — (DOCX) [file pone.0142902.s001.docx]

**S1 Appendix**

**SUPPLEMENTAL METHODS**

**Reagents**

Simvastatin (Cat. No. 567020) was purchased from Merck Millipore. GGTI 298 (Cat. No. 2430), FTI 277 (Cat. No. 2407), GW9508 (Cat. No. 2649), AS1269574 (Cat. No. 4177), GLP1 (7-36) amide (Cat. No. 2082), Pravastatin sodium salt (Cat. No. 2318), Forskolin (Cat. No. 1099), H89 dihydrochloride (Cat. No. 2910), 2-APB (Cat. No. 1224) and Caffeine (Cat. No. 2793) were purchased from TOCRIS Bioscience. Mevalolactone (Cat. No. M4667), GGPP (Geranylgeranyl pyrophosphate ammonium salt) (Cat. No. G6025), FPP (Farnesyl pyrophosphate ammonium salt) (Cat. No. F6892), KCl (P5405), Acetylcholine chloride (Cat. No. A2661), Neostigmine Bromide (Cat. No. N2001), Exendin-4 (Cat. No. E7144), Diazoxide (Cat. No. D9035), Nifedipine (Cat. No. N7634), Oleic acid (Cat. No. O1257), Linoleic acid-Water Soluble (Cat. No. L5900) and Tolbutamide (Cat. No.T0891) were from Sigma-Aldrich. BAPTA, AM (Cat. No. B1205) and EGTA, AM (Cat. No. E1219) were from Life Technologies. TAK-875 (Cat. No. S2637) was purchased from Selleckchem. PMA (Phorbol-12-myristate-13-acetate) (Cat. No. 524400) was purchased from Calbiochem. 8-Br-cAMP (Cat. No. B 007-100), 8-pCPT-2’-O-Me-cAMP (Cat. No. C 041-05) and ESI-05 (4- Methylphenyl- 2, 4, 6- trimethylphenylsulfone) (Cat. No. M092) were from BIOLOG Life Science Institute. All the chemicals used for preparing KRBH were purchased from Sigma-Aldrich, Germany.

**MIN6 cell culture**

MIN6 cells were kindly gifted by Merja Roivainen, National Institute for Health and Welfare, Helsinki, Finland, originally from Prof. Jun-ichi Miyazaki, Osaka University, Japan. Cells were cultured in DMEM containing 25 mM glucose supplemented with 15% heat inactivated fetal bovine serum (GIBCO), 2 mM L-glutamine (Lonza) and 100 units/ml penicillin, 100 µg/ml streptomycin (Lonza), 5 µl/l β-mercaptoethanol and 3.4 g/l NaHCO_3_. The cells were cultured at 37°C in a humidified atmosphere with 5 % CO_2_.

**Insulin secretion assay**

MIN6 cells were plated and cultured in 24 well plates. When the cells were confluent, insulin secretion assay was performed. Briefly, the cells were washed with glucose-free KRBH (Krebs-Ringer bicarbonate HEPES Buffer) (119 mM NaCl, 4.74 mM KCl, 2.54 mM CaCl_2_, 1.19 mM MgSO_4_, 1.19 mM KH_2_PO_4_, 10 mM HEPES, 25 mM NaHCO_3_ and 0.1% BSA at pH 7.4) for three times before incubating them in the same buffer at 37°C for 1 hour. After 1 hour, cells were washed once with glucose free KRBH and pre-incubated for 30 minutes with glucose free KRBH containing simvastatin (14.3 µM or 6 µg/ml), pravastatin (12 µg/ml), GLP-1 (100 nM), exendin-4 (20 nM), GGTI 298 (20 µM), FTI 277 (20 µM), mevalolactone (1 mM), GGPP (geranylgeranyl pyrophosphate ammonium salt) (20 µM), FPP (farnesyl pyrophosphate ammonium salt) (20 µM), TAK875 (40 µM), AS1269574 (40 µM), GW-9508 (40 µM), EGTA-AM (100 µM), BAPTA-AM (100 µM), 2-APB (2-aminoethoxydiphenylborane) (100 µM), nifedipine (5 µM), ESI-05 (20 µM), H89 (10 µM) and diazoxide (250 µM). In the experiments involving acetylcholine treatment, pre-incubation was done with neostigmine bromide (10 µM) to reversibly inhibit acetylcholinesterase which is an enzyme that deactivates acetyl choline. Due to this inhibition of acetylcholinesterase with neostigmine bromide, acetylcholine is not destroyed quickly and hence remains active for a longer time. In all the remaining 30 minute pre-incubation treatments, the cells were treated with only glucose-free KRBH. After this pre-incubation period, the cells were treated with compounds mentioned above in the same concentration and also with compounds tolbutamide (100 µM), acetylcholine (10 µM), PMA (phorbol-12-myristate-13-acetate) (0.5 µM), KCl (40 mM), 8-bromo-camp (1 mM), 8-pCPT-2’-O-Me-cAMP (100 µM), caffeine (10 mM), Rho/Rac/Cdc42 activator I (1, 2.5, 5 µg/ml), oleic acid (10 µM, 30 µM, 0.5 mM, 1 mM), linoleic acid (0.5 mM) and forskolin (20 µM) in KRBH containing either 5.5 mM or 16.7 mM glucose at 37°C for 1 hour in the experiments involving the respective compounds.

KRBH buffer from the wells was collected for insulin assay and the cells were washed with PBS once. Lysates from the respective wells were lysed with RIPA buffer containing protease and phosphatase inhibitors and collected for protein estimation and western blotting and stored at -70⁰C until the assays were performed. Protein estimation was determined with Pierce BCA protein assay kit (Cat. No. 23225, Pierce).

Insulin was measured by using AlphaLISA Insulin Kit (Cat. No. AL204C, PerkinElmer) according to the manufacturer’s instructions. Briefly, the samples were centrifuged for 30 minutes the supernatant was collected and stored at -70°C until the assay was performed. For the assay, 5 µl of supernatant of each sample was added in triplicates to each well of 384 well plate (Optiplate-384, White, Cat. No. 6007290; PerkinElmer) and to this, 20 µl of a 2.5X mixture of AlphaLISA Anti-Analyte Acceptor beads (10 µg/ml final concentration) and Biotinylated Antibody Anti-Analyte (1 nM final concentration) were added and incubated at 23⁰C for 60 minutes. After this time, 25 µl of 2X SA-Donor beads (40 µg/ml final concentration) was added to each well and the plate was incubated at 23°C for 60 minutes in the dark.

The results were obtained after correlating with the standard curve from human insulin analyte standard dilutions by the EnVision 2104 Multilabel Reader (Perkin Elmer). Insulin secretion levels were normalized with total protein concentration. Data presented are means ± SEM. p-values were calculated with Mann-Whitney test. Each group has 8 replicates.

**Glucose uptake assay**

MIN6 cells were plated in 24 well plates and cultured for 3 to 4 days until confluent. MIN6 cells were washed 3 times with glucose free KRBH buffer. After washing, the cells were pre-incubated with glucose free KRBH buffer for 1 hour. After 1 hour cells were treated with simvastatin (6 µg/ml or 14.3 µM) in the same buffer for 30 minutes. After 30 minutes the cells were washed once with glucose free KRBH buffer and treated with simvastatin (6 µg/ml or 14.3 µM) with KRBH buffer containing 5.5 mM glucose or 16.7 mM glucose for 1 hour. After the treatments as indicated in the experiments, KRBH buffer was removed and the cells were treated with glucose cocktail in KRBH buffer containing 0.2 mM glucose and 1 µCi 2-Deoxy-D-[2,6-^3^H]glucose (Cat. No. NET549250UC; Perkin Elmer, Boston, USA) and incubated for 15 minutes at room temperature. After 15 minutes the reaction was stopped by keeping the plates on ice and washing cells with ice cold PBS. 200 µl of 0.2 N NaOH was added to each well and the plate was incubated for 90 minutes at room temperature with constant shaking. The samples were collected and stored at -70°C until measured. Optiphase 2 was added to the samples and radioactivity was measured using the 1450 MicroBeta Trilux (Wallac). The glucose uptake was normalized with protein concentrations. p-values were calculated with Mann-Whitney test. Each group has 6 replicates.

**Immunoblotting**

Proteins were extracted using RIPA buffer along with protease inhibitors and phosphatase inhibitors (Roche). Protein concentrations were measured by BCA protein assay kit (Cat. No. 23225, Pierce, Rockford, USA). 20 μg/lane of protein samples containing NuPAGE LDS sample buffer and reducing agent were loaded into 4-12% NuPAGE Bis-Tris gels (Cat. No. NP0336BOX, Life Technologies Europe BV, Espoo, Finland), subjected to gel electrophoresis and transferred to polyvinylidene fluoride (PVDF) membranes (RPN303F, GE Healthcare Bio-sciences AB, Uppsala, Sweden).

For GLP-1R, phospho-AKT, AKT, glucose transporter 2 (GLUT2), phospho-insulin receptor, insulin receptor, insulin receptor substrate 1 (IRS1), phospho-IRS1, IRS2 and Epac2 proteins, membranes were blocked in 5% BSA, TBS 0.1% Tween-20, for 1 hour at room temperature, washed with TBS-0.1%Tween-20 for 3x5 minutes and incubated overnight at +4⁰C with respective primary antibodies (1:1000). For PKA proteins, membranes were blocked in 5% milk, TBS 0.1% Tween-20, for 1 hour at room temperature, washed with TBS-0.1%Tween-20 for 3x5 minutes and incubated at +4⁰C overnight with PKAα catalytic or PKA 1β regulatory antibodies. After the primary antibody incubations, the membranes were washed with TBS-0.1% Tween-20 for 3x5 minutes before incubating the membranes with secondary anti-rabbit horseradish peroxidase–conjugated immunoglobulin (Cat. No. NA934V, GE Healthcare) (1:10000) for 1 hour at room temperature. The membranes were finally washed with TBS-0.1% Tween-20 for 3x5 minutes and TBS for 5 minutes.

For α-tubulin proteins, the membranes were blocked in 3% milk in PBS-0.05% Tween-20 for 1 hour, incubated with primary antibody dilutions (1:10000 in blocking buffer) for 1 hour and finally incubated with secondary anti-mouse horseradish peroxidase-conjugated immunoglobulin (Cat. No. NA931V; GE Healthcare) (1:20000 in blocking buffer) for 1 hour. All these incubations were conducted at room temperature and were washed with PBS-0.05% Tween-20 for 3x5 minutes in between all the incubations and after the secondary antibody incubation. For actin proteins, the membranes were blocked in PBS with 3% milk and 0.05% Tween-20 for 1 hour at room temperature, washed with PBS-0.05% Tween-20 for 3x5 minutes and incubated with actin primary antibodies (1:100) for 2 hours at room temperature. The membranes were washed with PBS-0.05% Tween-20 for 3x5 minutes before incubating them with secondary anti-goat horseradish peroxidase–conjugated immunoglobulin (Cat. No. sc-2020, Santa Cruz Biotechnology) (1:2000) in PBS-0.05% Tween-20 for 2 hours at room temperature. For GAPDH proteins, the membranes were blocked in 10% milk in 0.1% TBS-Tween for 2 hours at room temperature, incubated with primary antibody dilutions (1:5000 in blocking buffer) overnight at +4⁰C and finally incubated with secondary anti-mouse horseradish peroxidase–conjugated immunoglobulin (Cat. No. NA931V; GE Healthcare) (1:10000 in blocking buffer) for 1 hour. The membranes were washed with 0.1% TBS-Tween for 3x5 minutes in between all the incubations and after the secondary antibody incubation.

The bands were visualized using chemiluminescence (ECL plus) and images were captured in Image Quant RT-ECL machine. Quantification of the bands was done by applying Quantity One software (Bio-Rad). The protein expressions presented were normalized with either α-tubulin or GAPDH or actin protein levels as shown in the corresponding figures.

Phospho-AKT (Ser473) (Cat. No. 9271), AKT (Cat. No. 9272), insulin receptor β (4B8) (Cat. No. 3025), phospho-IRS1 (Cat. No. 3203), IRS1 (D23G12) (Cat. No. 3407) and IRS2 (Cat. No. 4502) were purchased from Cell Signaling. GLP-1R (Cat. No. ab39072), phospho-insulin receptor (Cat. No. ab60946) and GAPDH (Cat. No.ab8245) were purchased from Abcam. α-tubulin (Cat. No.T5168) was bought from Sigma-Aldrich. GLUT2 (H-67): (sc-9117), Epac2 (H-220) (Cat. No. sc-25633), PKAα catalytic (c-20) (Cat. No. sc-903), PKA1β regulatory (C-19) (Cat. No. sc-907) and actin (I-19) (Cat. No. sc-1616) were purchased from Santa Cruz Biotechnology.

**Pyruvate assay**

Pyruvate levels from MIN6 cells were measured with Pyruvate assay kit (Cat. No. ab65342, Abcam). Briefly MIN6 cells were grown in 96 well clear plates and the confluent cells were washed 3 times with glucose free KRBH and then incubated with glucose free KRBH for 1 hour. The cells were then washed once and pre-incubated with simvastatin (14.3 µM) in glucose free KRBH for 30 minutes and later incubated with simvastatin (14.3 µM) in KRBH containing 5.5 mM glucose concentration for 1 hour. After this incubation, colorimetric assay was performed and pyruvate was measured at OD570nm according to the manufacturer’s instructions. Samples were deproteinized by 10 kDa cutoff spin filter to remove proteins. Standard curve was plotted with nmol/well and OD570nm readings. Sample readings were applied to the standard curve after subtracting the background from zero pyruvate control reading from all sample readings. Each group has 6 replicates.

**ADP/ATP ratio assay**

ADP/ATP ratio of MIN6 cells was determined with an ADP/ATP Ratio Assay kit (Cat. No. ab65313, Abcam). 1x10^4^ MIN6 cells were plated in each well of 96 well plates and after they were confluent, the cells were washed 3 times with glucose free KRBH and then incubated with glucose free KRBH for 1 hour. The cells were then washed once and pre-incubated with simvastatin (14.3 µM) in glucose free KRBH for 30 minutes and later incubated with simvastatin (14.3 µM) in KRBH containing 5.5 mM and 16.7 mM glucose concentrations for 1 hour. The ADP/ATP ratio was measured according to the instructions given by the manufacturer. Briefly, 100 µl of reaction mix was added to each well of a 96 well luminometer plate and the background luminescence was read. KRBH buffer was removed and cells were treated with nucleotide releasing buffer for 5 minutes at room temperature with gentle shaking, transferred into luminometer plate and the luminescence was measured in a luminometer after 5 minutes (Data A). The samples were measured after 10 minutes (Data B) and then after adding 1 µl ADP converting enzyme, the samples were again read in 5-10 minutes in a luminometer (Data C). ADP/ATP ratio was calculated as Data C – Data B/ Data A. Each group has 6 replicates.
