# Supplementary figures and images for: Simvastatin Impairs Insulin Secretion by Multiple Mechanisms in MIN6 Cells
Source: PLoS One. 2015 Nov 11;10(11):e0142902. doi: 10.1371/journal.pone.0142902 (PMC4641640; doi:10.1371/journal.pone.0142902)

**S2 Appendix**

**FULL BLOT INFORMATION FOR SUPPLEMENTAL FIGURES S1-3**

**S1 FIGURE**

**
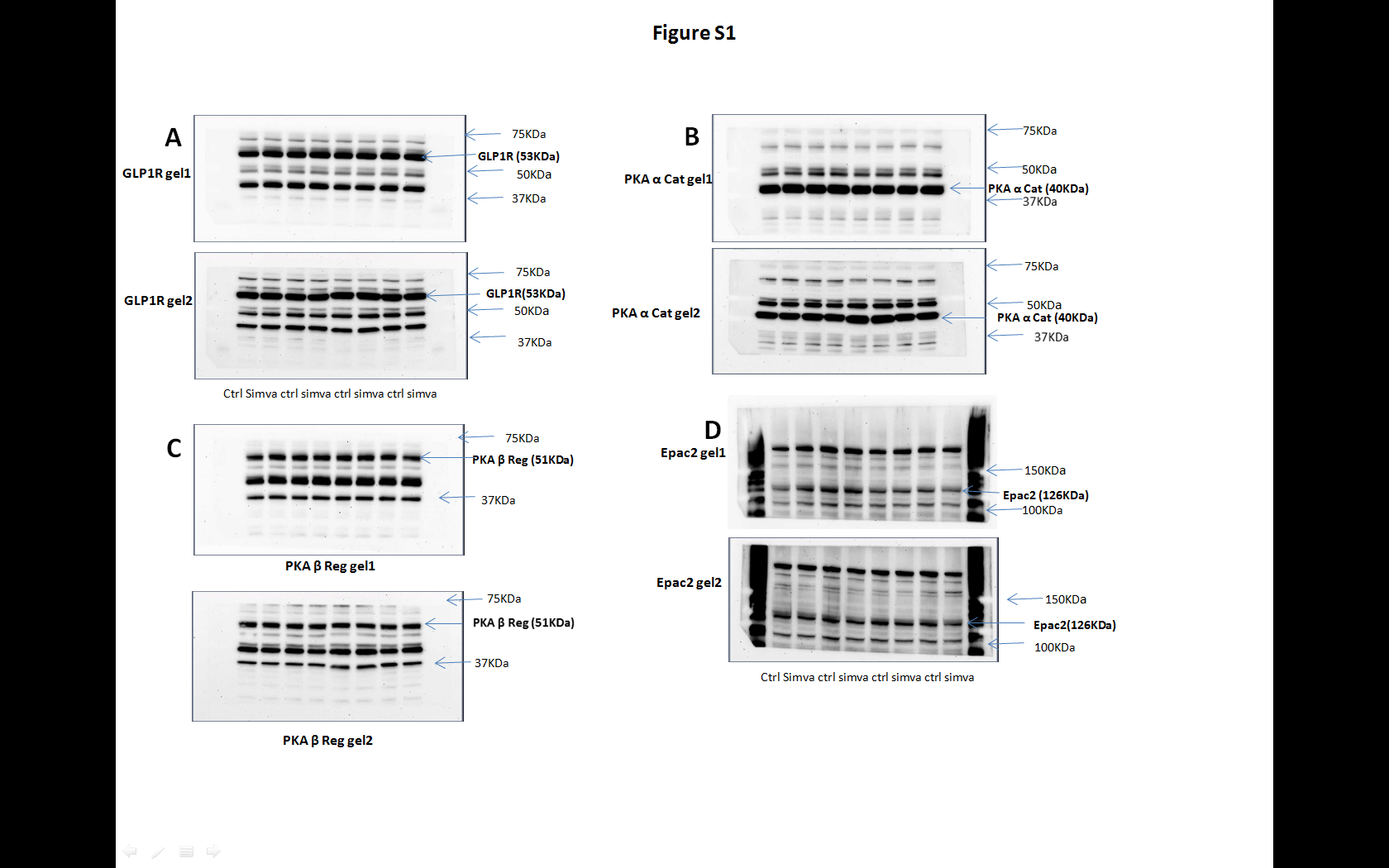
**

**
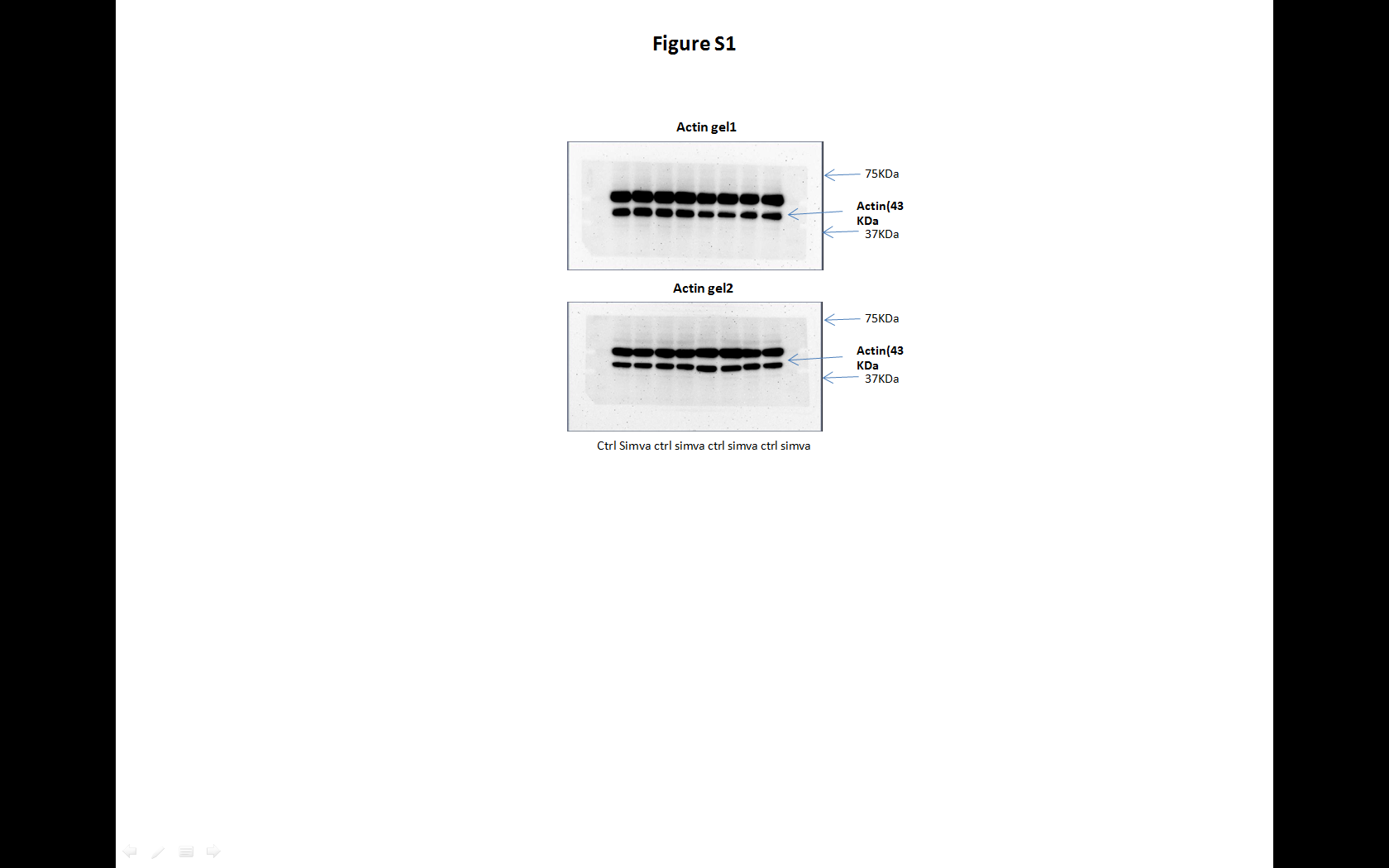
**

**S2 FIGURE**

**
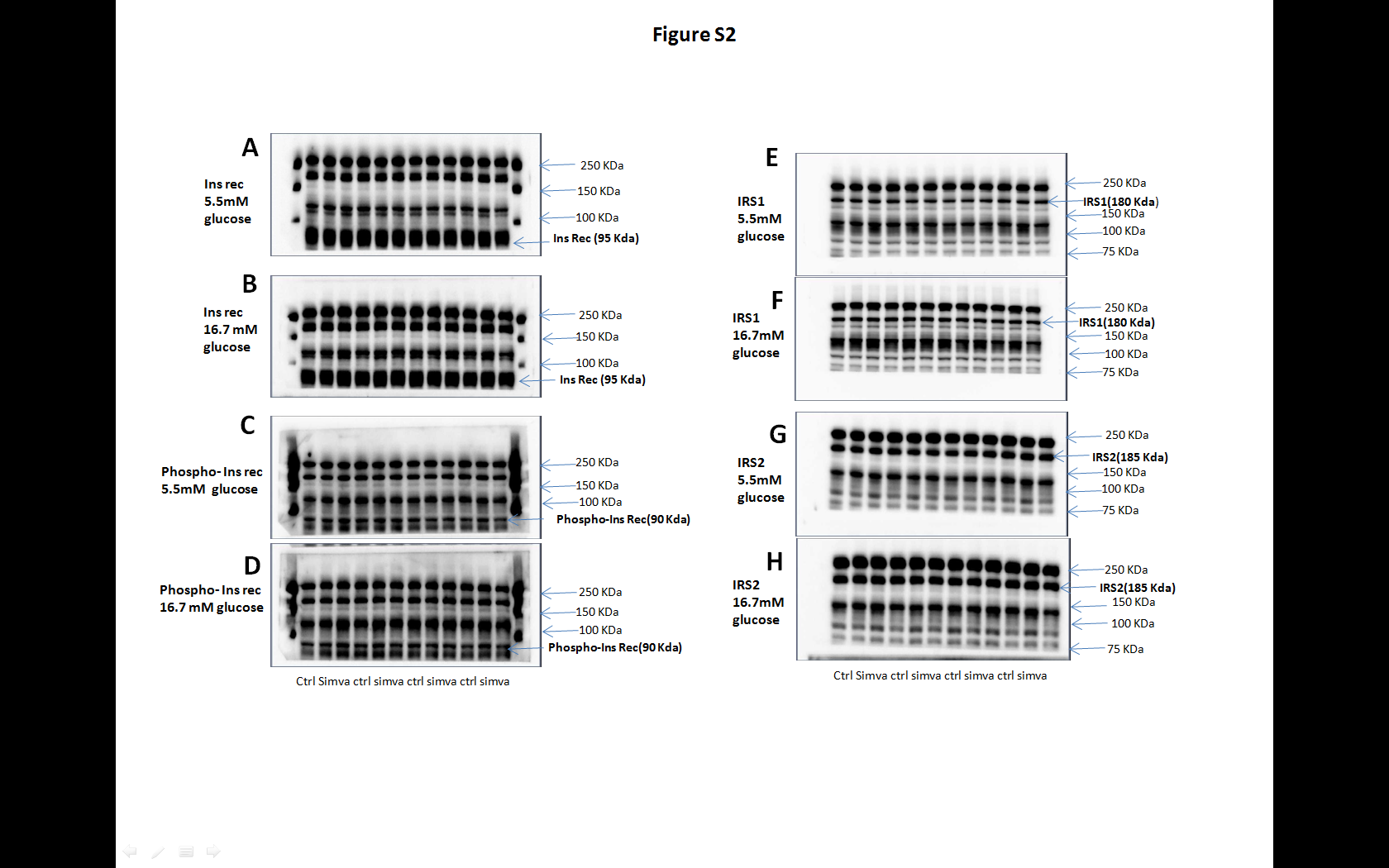
**

**
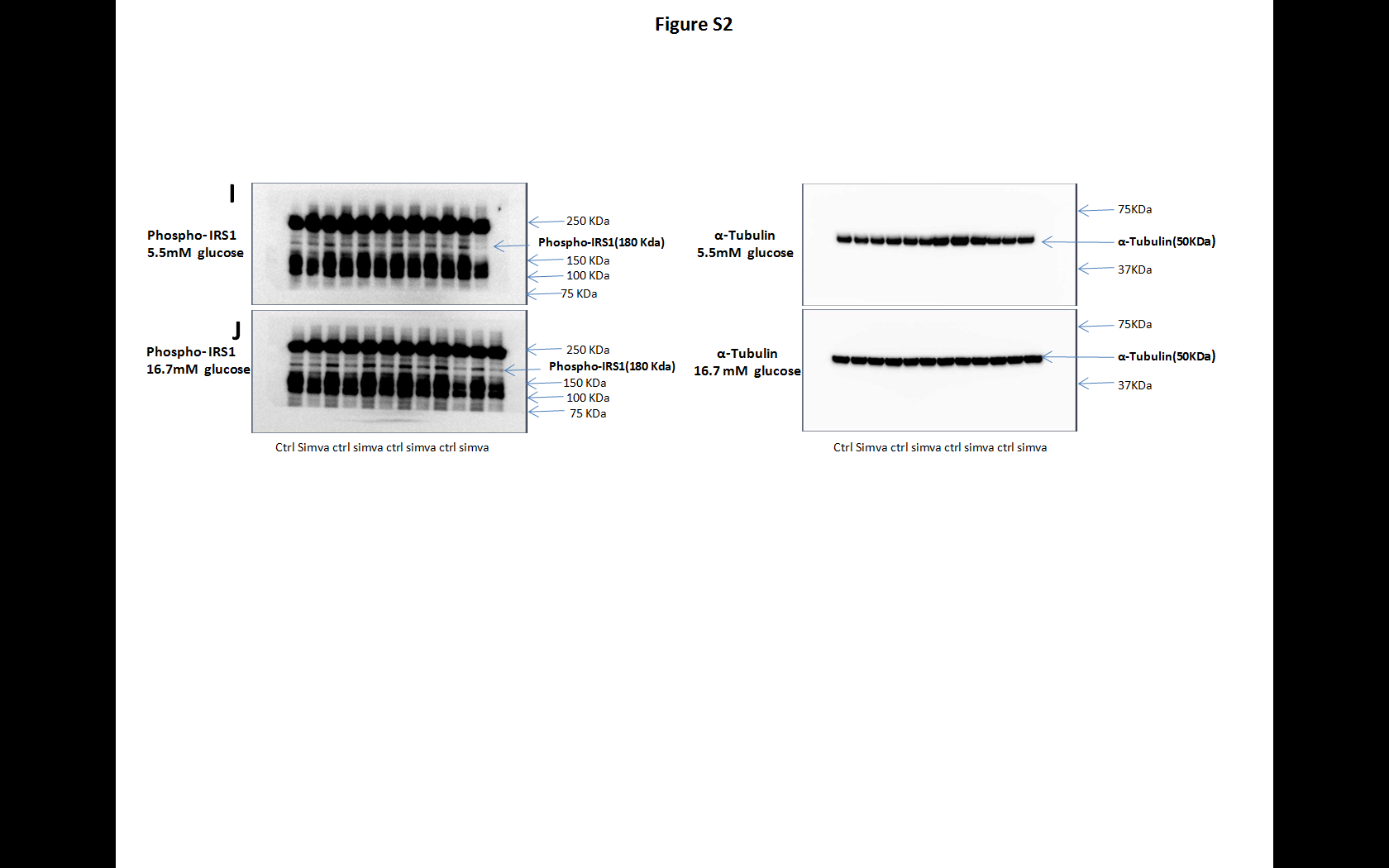
**

**S3 FIGURE**

**
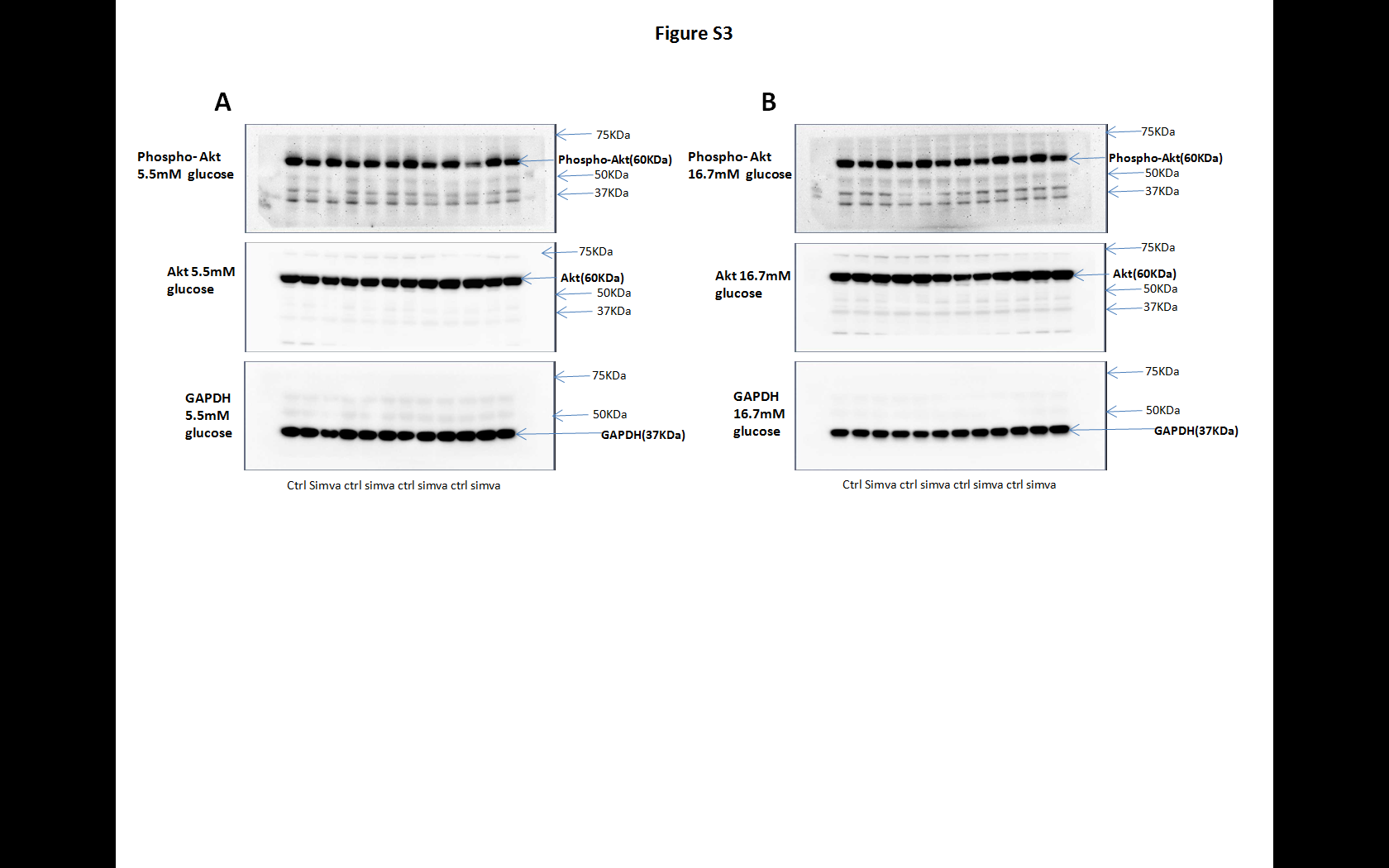
**

**
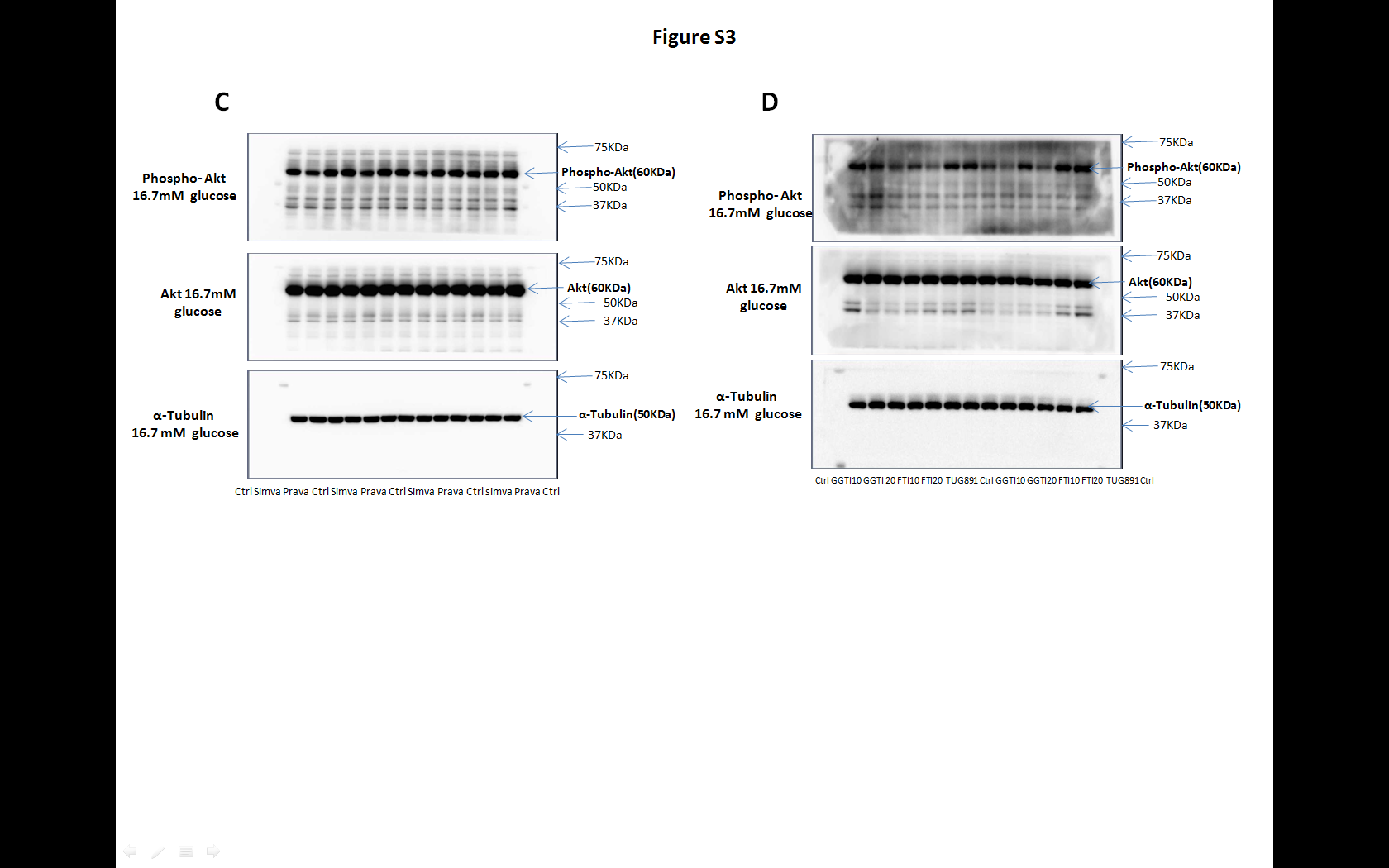
**

**
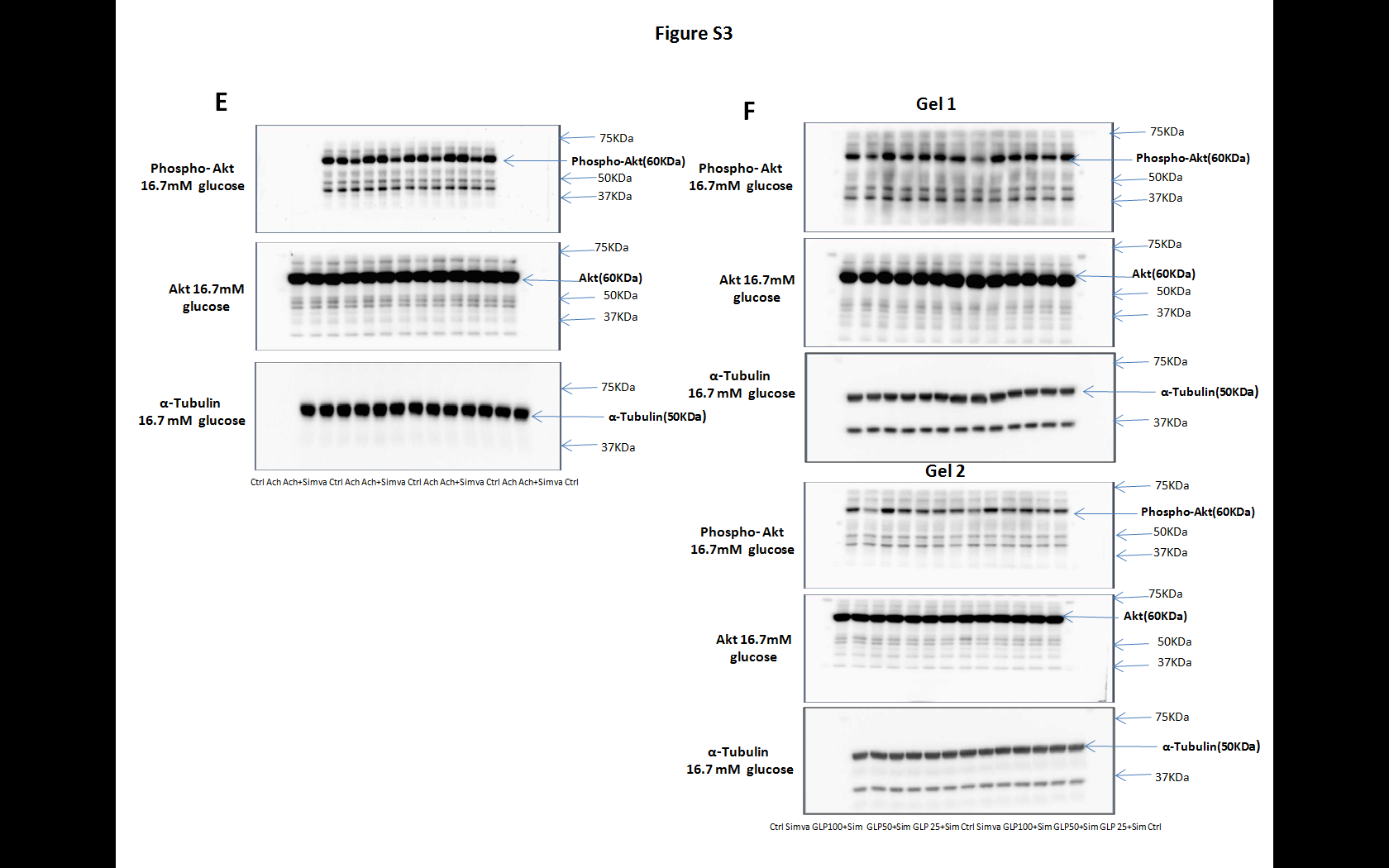
**

Supplement: S2 Appendix — (DOCX) [file pone.0142902.s002.docx]
