# Supplementary material for: Simvastatin Impairs Insulin Secretion by Multiple Mechanisms in MIN6 Cells
Source: PLoS One. 2015 Nov 11;10(11):e0142902. doi: 10.1371/journal.pone.0142902 (PMC4641640; doi:10.1371/journal.pone.0142902)
Supplement: S1 Table — (DOCX) [file pone.0142902.s004.docx]

**S1 Table.** List of cells, reagents, materials, assays, antibodies and other equipment used for the experiments and their sources.

| **Item** | **Cat. No.** | **Source** |
| --- | --- | --- |
| **Cells** |  |  |
| MIN6 cells |  | Merja Roivainen, National Institute for Health and Welfare, Helsinki, Finland; originally from Prof. Jun-ichi Miyazaki, Osaka University, Japan |
| GPR119-overexpressing CHO-K1 cells |  | Euroscreen |
| GPR40-overexpressing CHO-K1-mt aequorin cells |  | Euroscreen |
| **Reagents and materials** |  |  |
| 2-APB | 1224 | TOCRIS bioscience |
| 2-Deoxy-D-[2,6-^3^H] glucose | NET549250UC | Perkin Elmer |
| 2-Mercaptoethanol | M3148 | Sigma-Aldrich |
| 4-12% NuPAGE Bis-Tris gels | NP0336BOX | Life Technologies |
| 8-Br-cAMP | B007-100 | BIOLOG Life Science Institute |
| 8-pCPT-2’-O-Me-cAMP | C041-05 | BIOLOG Life Science Institute |
| Acetylcholine chloride | A2661 | Sigma-Aldrich |
| AS1269574 | 4177 | TOCRIS bioscience |
| BAPTA, AM | B1205 | Life Technologies |
| Caffeine | 2793 | TOCRIS bioscience |
| Chemicals for preparing KRBH | - | Sigma-Aldrich |
| Diazoxide | D9035 | Sigma-Aldrich |
| DMEM | BE12-614F | Lonza |
| EGTA, AM | E1219 | Life Technologies |
| ESI-05 (4- Methylphenyl- 2, 4, 6- trimethylphenylsulfone) | M092 | BIOLOG Life Science Institute |
| Exendin-4 | E7144 | Sigma-Aldrich |
| FBS | 10500-064 | Gibco, Life Technologies |
| Forskolin | 1099 | TOCRIS bioscience |
| FPP (Farnesyl pyrophosphate ammonium salt) | F6892 | Sigma-Aldrich |
| FTI-277 | 2407 | TOCRIS bioscience |
| GGPP (Geranylgeranyl pyrophosphate ammonium salt) | G6025 | Sigma-Aldrich |
| GGTI-298 | 2430 | TOCRIS bioscience |
| GLP1 (7-36) amide | 2082 | TOCRIS bioscience |
| GW9508 | 2649 | TOCRIS bioscience |
| H89 dihydrochloride | 2910 | TOCRIS bioscience |
| KCl | P5405 | Sigma-Aldrich |
| L-Glutamine | BE17-605E | Lonza |
| Linoleic acid-Water Soluble | L5900 | Sigma-Aldrich |
| Mevalonolactone | M4667 | Sigma-Aldrich |
| Neostigmine Bromide | N2001 | Sigma-Aldrich |
| Nifedipine | N7634 | Sigma-Aldrich |
| Oleic acid | O1257 | Sigma-Aldrich |
| Optiplate-384 | 6007290 | PerkinElmer |
| Pen-Strep | DE17-602E | Lonza |
| Phosphatase inhibitors | 4906837001 | Roche |
| PMA (Phorbol-12-myristate-13-acetate) | 524400 | Calbiochem |
| Polyvinylidene fluoride (PVDF) membranes | RPN303F | GE Healthcare |
| Pravastatin sodium salt | 2318 | TOCRIS bioscience |
| Protease inhibitors | 11697498001 | Roche |
| Rho/Rac/Cdc42 activator I | CN04-A | Cytoskeleton |
| RIPA buffer | 89901 | Pierce |
| Simvastatin | 567020 | Merck Millipore |
| TAK-875 | S2637 | Selleckchem |
| Tolbutamide | T0891 | Sigma-Aldrich |
| **Assays** |  |  |
| ADP/ATP ratio assay kit | ab65313 | Abcam |
| AlphaLISA Insulin Kit | AL204C | PerkinElmer |
| Pierce BCA protein assay | 23225 | Pierce |
| Pyruvate assay kit | ab65342 | Abcam |
| **Antibodies** |  |  |
| AKT | 9272 | Cell Signaling |
| Epac2 (H-220) | sc-25633 | SantaCruz Biotechnology |
| GAPDH | ab8245 | Abcam |
| GLP-1R | ab39072 | Abcam |
| GLUT2 (H-67) | sc-9117 | SantaCruz Biotechnology |
| Insulin receptor β (4B8) | 3025 | Cell Signaling |
| IRS1 (D23G12) | 3407 | Cell Signaling |
| IRS2 | 4502 | Cell Signaling |
| Phospho-AKT (Ser473) | 9271 | Cell Signaling |
| Phospho-insulin receptor | ab60946 | Abcam |
| Phospho-IRS1 | 3203 | Cell Signaling |
| PKA1β regulatory (C-19) | sc-907 | SantaCruz Biotechnology |
| PKAα catalytic (c-20) | sc-903 | SantaCruz Biotechnology |
| Secondary anti-goat HRP-conjugated immunoglobulin | sc-2020 | SantaCruz Biotechnology |
| Secondary anti-mouse HRP-conjugated immunoglobulin | NA931V | GE Healthcare |
| Secondary anti-rabbit HRP-conjugated immunoglobulin | NA934V | GE Healthcare |
| Actin (I-19) | sc-1616 | SantaCruz Biotechnology |
| α-tubulin | T5168 | Sigma-Aldrich |
| **Other equipment** |  |  |
| EnVision 2104 Multilabel Reader | - | Perkin Elmer |
