# Supplementary material for: Simvastatin Impairs Insulin Secretion by Multiple Mechanisms in MIN6 Cells
Source: PLoS One. 2015 Nov 11;10(11):e0142902. doi: 10.1371/journal.pone.0142902 (PMC4641640; doi:10.1371/journal.pone.0142902)
Supplement: S1 Fig — (DOCX) [file pone.0142902.s005.docx]

**S1 Figure.** **Effect of simvastatin on protein expression of different proteins involved in** **GLP-1 signaling pathway in MIN6 β-cells:** The effect of simvastatin (Simva) (14.3 µM) treatment at 16.7 mM glucose concentration is shown on: glucagon like peptide-1 receptor (GLP1R) (**A**), protein kinase A alpha catalytic (PKA-α cat) (**B**), protein kinase A beta regulatory (PKA-β reg) (**C**) EPAC2 (**D**), and the corresponding western blots (**E**). Data are means (±SEM) relative to control (Ctrl) (100%). p-values were calculated with t-test. Each group has 8 replicates.
