# Supplementary material for: Simvastatin Impairs Insulin Secretion by Multiple Mechanisms in MIN6 Cells
Source: PLoS One. 2015 Nov 11;10(11):e0142902. doi: 10.1371/journal.pone.0142902 (PMC4641640; doi:10.1371/journal.pone.0142902)
Supplement: S2 Fig — (DOCX) [file pone.0142902.s006.docx]

**S2 Figure.** **Effect of simvastatin on protein expression of different proteins involved in insulin signaling pathway in MIN6 β-cells:** The effect of simvastatin (Simva) (14.3 µM) treatment at 5.5 mM and 16.7 mM glucose concentration respectively is shown on: total protein expression of insulin receptor (IR) (**A, B**), phosphorylation of insulin receptor (p-IR) (**C, D**), protein expression of insulin receptor substrate-1 (IRS1) (**E, F**) and insulin receptor substrate-2 (IRS2) (**G, H**), phosphorylation of IRS1 (p-IRS1) (**I, J**), and the corresponding western blots (**K, L**). Data are means (±SEM) relative to control (Ctrl) (100%). p-values were calculated with t-test. Each group has 6 replicates.
