# Supplementary material for: Simvastatin Impairs Insulin Secretion by Multiple Mechanisms in MIN6 Cells
Source: PLoS One. 2015 Nov 11;10(11):e0142902. doi: 10.1371/journal.pone.0142902 (PMC4641640; doi:10.1371/journal.pone.0142902)
Supplement: S3 Fig — (DOCX) [file pone.0142902.s007.docx]

**S3 Figure.** **Effect of simvastatin and other compounds on AKT activation and phosphorylation in** **MIN6 β-cells**: The effect of simvastatin (14.3 µM) (Simva) at 5.5 mM (**A**) and 16.7 mM (**B**) glucose concentration; the effect of pravastatin (Prava) (109.76 µM) at 16.7 mM glucose concentration (**C**); the effect of the inhibition of isoprenoid intermediates farnesyl pyrophosphate and geranylgeranyl pyrophosphate with farnesyltransferase inhibitor (FTI 277, 20 µM) and geranylgeranyltransferase inhibitor (GGTI 298, 20 µM) at 16.7 mM glucose concentration (**D**); the effect of acetylcholine (Ach, 10 µM) alone or in combination with simvastatin (Simva) at 16.7 mM glucose concentration (**E**); and the effect of GLP-1 at concentrations of 25 nM, 50 nM and 100 nM alone or in combination with simvastatin at 16.7 mM glucose concentration (**F**) on AKT phosphorylation; the effect of AKT inhibitors MK2206 (1 µM) and perifosine (20 µM) alone or in combination with simvastatin on insulin secretion at 16.7 mM glucose concentration (**G**), and the corresponding western blots are shown under each figure. Insulin secretion values are normalized with protein concentration. Data are means (±SEM) relative to control (Ctrl) (100%). p-values were calculated with t-test (A-F) or Mann-Whitney test (G), in F *p<0.05 compared to simvastatin treatment. Each group has 6 (A, B), 4 (C, E, F), 2 (D) or 5 (G) replicates.
